# Signaling of MK2 sustains robust AP1 activity for triple negative breast cancer tumorigenesis through direct phosphorylation of JAB1

**DOI:** 10.1038/s41523-021-00300-1

**Published:** 2021-07-09

**Authors:** Haoming Chen, Ravi Padia, Tao Li, Yue Li, Bin Li, Lingtao Jin, Shuang Huang

**Affiliations:** 1 The Ministry of Education Key Laboratory of Contemporary Anthropology, College of Life Science, Fudan University, Shanghai, China; 2 Department of Anatomy and Cell Biology, University of Florida College of Medicine, Gainesville, FL USA

**Keywords:** Cell signalling, Breast cancer

## Abstract

Triple negative breast cancer (TNBC) cells are generally more invasive than estrogen receptor-positive (ER + ) breast cancer cells. Consistent with the importance of activator protein 1 (AP1) transcription factors in invasion, AP1 activity is much higher in TNBC lines than ER + lines. In TNBC cells, robust AP1 activity is facilitated by both ERK and p38^MAPK^ signaling pathways. While ERK signaling pathway regulates AP1 activity by controlling the abundance of AP1 transcription factors, p38^MAPK^ signaling pathway does it by enhancing AP1 binding to AP1 sites without altering their abundance. Here, we show that p38^MAPK^ regulation of AP1 activity involves both MAPKAPK2 (MK2) and JAB1, a known JUN-binding protein. MK2 not only interacts with JAB1 but also directly phosphorylates JAB1 at Ser177 in TNBC cells. Interestingly, Ser177 phosphorylation does not affect JAB1 and JUN interaction. Instead, interfering with p38^MAPK^ signaling pathway or introducing an S to A point mutation at Ser177 of JAB1 reduces JUN recruitment to the AP1 sites in cyclin D1, urokinase plasminogen activator (uPA) and uPA receptor promoters. Moreover, knockdown of JAB1 diminishes >60% of AP1 transcriptional activity in TNBC cells. Taken together, these results indicate that MK2-mediated phosphorylation of JAB1 facilitates JUN recruitment to AP1 sites, thus augmenting AP1 activity. In line with the role of JAB1 in AP1 activity, silencing JAB1 leads to dramatic reduction in TNBC cell growth, in vitro invasion and in vivo tumor outgrowth. This study suggests that the p38^MAPK^-MK2 signaling pathway promotes TNBC tumorigenesis by sustaining robust AP1 activity.

## Introduction

Activator protein 1 (AP1) is a heterodimer or homodimer made up by proteins from the Jun and Fos families, and the closely related Atf and Maf families^1^. AP1 participates in almost all cellular functions, and is activated in response to a plethora of extracellular signals ranging from growth factors to stress and inflammation^2^. Dysregulated expression of Jun family members is frequent in diverse cancer types including breast^3^ and prostate cancer^4^. Increased AP1 activity has been reported in lung^5^, endometrial^6^, and cervical cancer^7^. In fact, chronic exposure to various carcinogens including tobacco smoke, asbestos, and ethanol upregulates the abundance of AP1 and induces AP1 activity^8–10^. Importantly, AP1 activity is crucial for tumorigenesis led by these carcinogens because dominant negative JUN mutant or AP1 decoys block tumor formation^11,12^. In addition, extensive evidences have also linked AP1 activity to cancer invasion and metastasis. For example, level of AP1 bound to DNA is higher in metastatic than less metastatic breast cancer cells and AP1 activity correlates well with invasiveness^13^. Especially, ectopically expressed JUN augments invasiveness of less invasive breast cancer cells while suppressing JUN expression inhibits invasion and metastasis^14^. We previously showed that high level of JUN and Fra-1 in triple negative breast cancer (TNBC) cells depends on ERK signaling pathway^15^. As the abundance of JUN and Fra-1 is generally associated with AP1 activity, ERK signaling pathway can be expected to promote AP1 activity by sustaining high expression of AP1 members. JNK and p38^MAPK^ signaling pathways have also been reported to promote AP1 activity through the phosphorylation of JUN and FOS^16,17^. Intriguingly, FOS is little detected in TNBC cells^15^ while the JNK activity has been found to be dispensable for AP1 induced by retinoid in breast cancer cells^18^. It remains unclear whether JNK and p38^MAPK^ signaling pathways contribute to sustained AP1 activity and tumorigenesis of breast cancer cells.

JUN activation domain-binding protein 1 (JAB1) was originally identified as a transcriptional coactivator of JUN and Jun D by stabilizing AP1 complex with AP-1 binding sites^19^. Subsequent studies also revealed that JAB1 participates in developmental regulation as the fifth component of the COP9 signalosome complex (CSN) which is independent of its role as an AP1 coactivator^20^. Several recent studies present an oncogenic role of JAB1 in various cancer types. For example, JAB1 interacts with S100A7 to promote cell survival through the enhancement of NF-κB and Akt activities in breast cancer cells^21^. Moreover, JAB1 also inactivates tumor suppressors including p53, SMAD4, and p27 by altering their subcellular localization or protein stabilities^22–25^. However, the importance of JAB1-AP1 interaction in the context of cancer cell growth and invasion remains to be investigated.

In this study, we have linked p38^MAPK^-MAPKAPK2 (MK2) signaling pathway to robust AP1 activity and TNBC tumorigenesis. MK2 phosphorylates JAB1 at Ser177 and MK2-mediated phosphorylation of JAB1 enhances JUN recruitment to AP1 binding sites, thus promoting AP1 activity in TNBC cells. Consistent to the role of JAB1 in AP1 activity, knockdown of JAB1 displayed reduced cell growth, in vitro invasion and in vivo tumor outgrowth. Our results suggest that the p38^MAPK^-MK2 signaling pathway promotes TNBC tumorigenesis by sustaining AP1 activity through Ser177 phosphorylation of JAB1.

## Results

### The p38^MAPK^-MK2 signaling pathway contributes to robust AP1 activity in TNBC cells

**Fig. 1:**
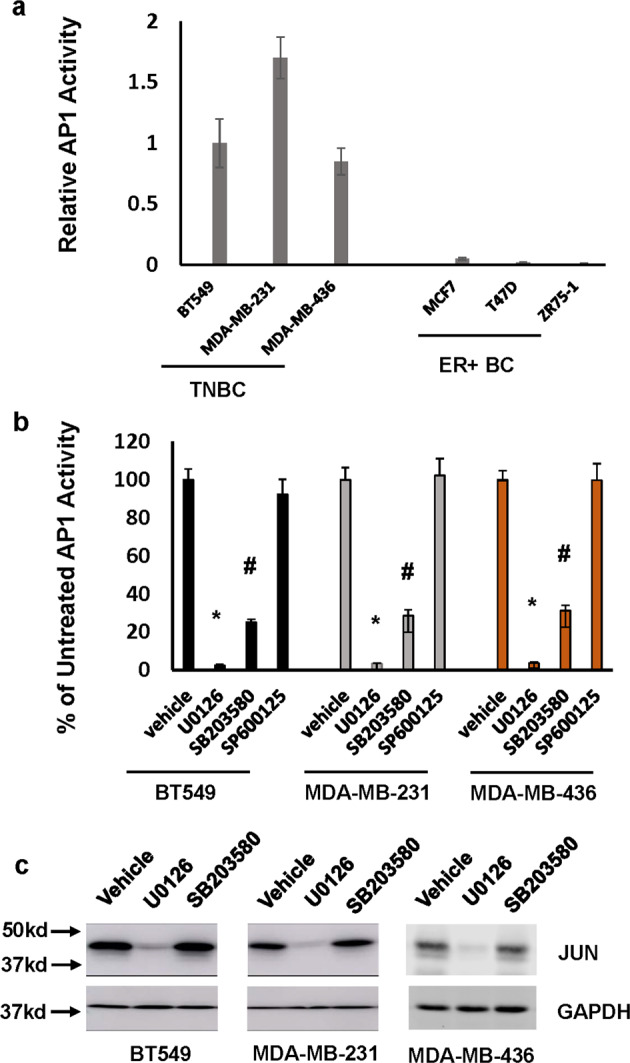
Both ERK and p38^MAPK^ signaling pathways contribute to sustained AP1 activity in TNBC cells. **a** Endogenous AP1 activity in breast cancer cell lines analyzed by AP1 site-containing luciferase reporter plasmid. Data are means ± SD from three experiments. **b** BT549, MDA-MB-231, and MDA-MB-436 cells were treated with 5 µM U0126, SB203580, SP600125, or vehicle for 24 h followed by analyzing AP1 activity. Data are means ± SD from three experiments. *P < 0.001 vs vehicle; #*P* < 0.01 vs vehicle. **c** BT549, MDA-MB-231, and MDA-MB-436 cells were treated with 5 µM U0126, SB203580, or vehicle for 24 h followed by western analysis to detect JUN and GAPDH with the respective antibodies. Data are the representative of three independent experiments. All blots derived from the same experiment and were processed in parallel.

AP1 activity has long been recognizes as a key factor impacting cancer cell growth and invasion. With the aid of AP1 luciferase reporter gene construct, we detected high AP1 activity in all TNBC lines while little AP1 activity was present in ER + lines (Fig. 1a). To determine the impact of various MAPK signaling pathways on AP1 activity in TNBC cells, we treated AP1 reporter construct-transfected BT549, MDA-MB-231, and MDA-MB-436 cells with MEK1/2 inhibitor U0126, p38^MAPK^ inhibitor SB203580 or JNK inhibitor SP600125 for 24 h followed by measuring the luciferase activity. While U0126 almost abolished AP1 activity, SB203580 reduced >70% of AP1 activity and the effect of SP600125 on AP1 activity was not apparent (Fig. 1b). The blocking effect of U0126 on AP1 activity can be readily explained by the observation that JUN disappeared in U0126-treated TNBC cells (Fig. 1c). However, the treatment of SB203580 did not alter its abundance in TNBC cells (Fig. 1c), indicating that p38^MAPK^ signaling pathway regulates AP1 activity in a mechanism independent of regulating expression of JUN.

**Fig. 2:**
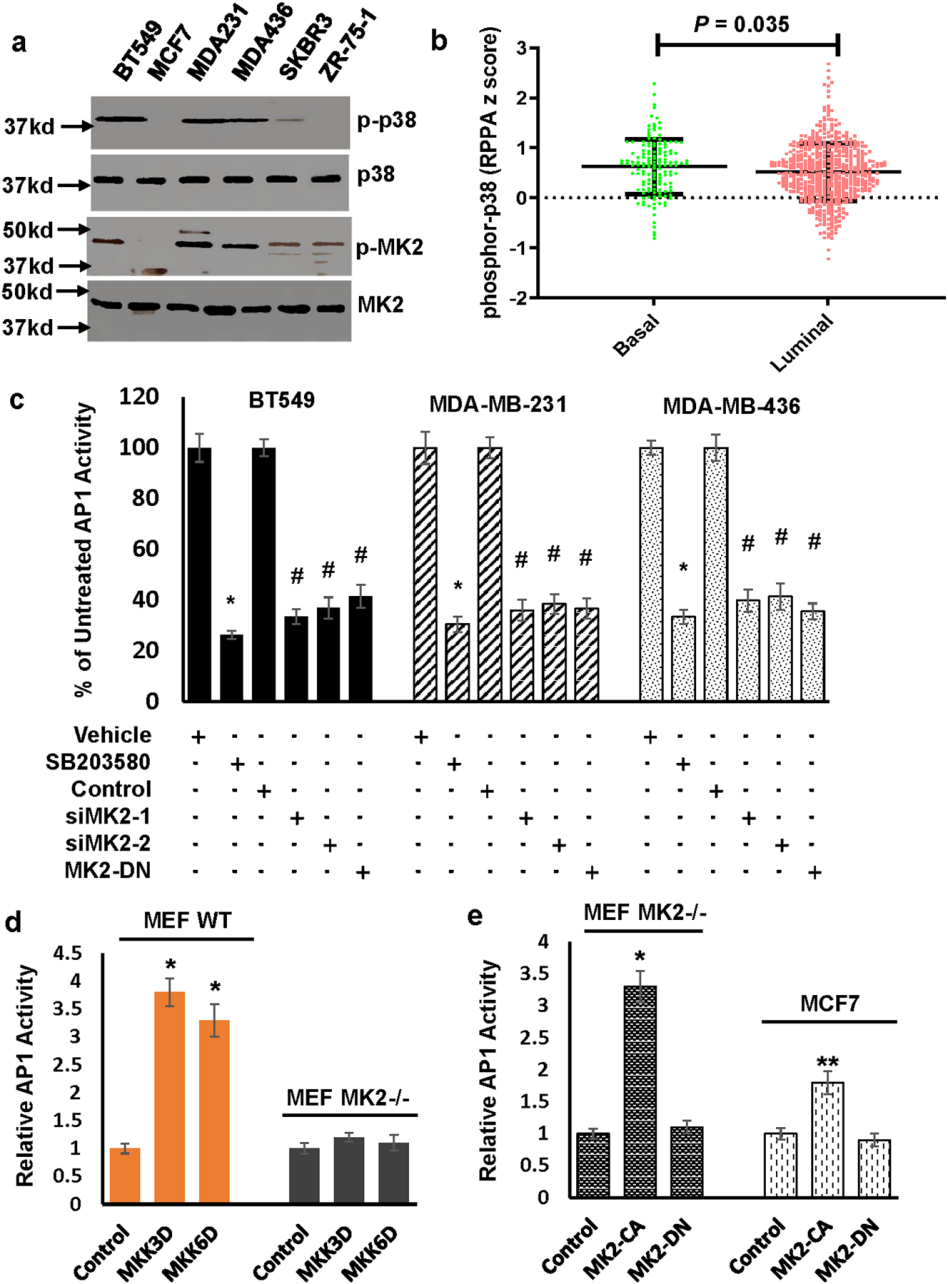
p38MAPK signaling pathway regulates AP1 activity in an MK2-dependent manner. **a** Overnight-culture cells were collected for western blot analysis to detect phosphor-p38^MAPK^ (p-p38), p38^MAPK^, phosphor-MK2 (p-MK2), and MK2 with the respective antibodies. Data are the representative of three independent experiments. All blots derived from the same experiment and were processed in parallel. **b** RPPA *z* score and corresponding clinical data from TCGA were used to analyze the level phosphor-p38^MAPK^ (pT180/Y182) and the comparison was made using GraphPad Prime 7.0 with unpaired *t* test (two-tailed). **c** BT549, MDA-MB-231 or MDA-MB-436 cells were treated with 5 µM SB203580 for 24 h or 30 nM MK2 siRNA (siMK2-1 and siMK2-2) for 3 days followed by analyzing AP1 activity. Vehicle was used for comparison of SB203580 treatment while scrambled siRNA control was used for comparison of MK2 siRNAs. Data are means ± SD from three experiments. *P < 0.005 vs vehicle; #*P* < 0.01 vs Control. **d** Wild-type or MK2−/− MEFs are transduced with MKK3D, MKK6D, or empty vector for 3 days followed by analyzing AP1 activity. Data are means ± SD from three experiments. *P < 0.005 vs Control. **e** MK2−/− MEFs or MCF7 cells are transduced with MK2-CA, MK2-DN, or empty vector for 3 days followed by analyzing AP1 activity. Data are means ± SD from three experiments. *P < 0.005 vs Control. **P < 0.05.

To investigate the link between AP1 Activity and p38^MPAK^ signaling pathway, we first examined p38^MAPK^ activity by detecting phosphorylated p38^MAPK^ in both TNBC and ER + cell lines. The level of phosphorylated p38^MAPK^ was readily detected in TNBC lines while was very low or undetectable in ER + lines (Fig. 2a). This observation is consistent with the analysis of human breast cancer Reverse Phase Protein Array dataset in which the level of phosphorylated p38^MAPK^ was higher in basal-like breast cancer (overwhelmingly overlapping with TNBC) than luminal subtype (ER + breast cancer) (Fig. 2b). Moreover, the level of phosphorylated MK2 (a major downstream kinase of p38^MAPK^) was similarly higher in TNBC lines than ER + ones (Fig. 2a). To explore the potential role of MK2 in p38^MAPK^ regulation of AP1 activity, we showed that knockdown of MK2 or ectopically expressing dominant negative MK2 (MK2-DN) suppressed AP1 activity to an extent similar to SB203580 (Fig. 2c and Supplementary Fig. 1). Consistent with the observed effect of MK2 knockdown and forced dominant negative MK2 expression on AP1 activity in TNBC cells, constitutively active MKK3 and MKK6, two upstream kinases of p38^MAPK^ increased AP1 activity in wild-type murine embryonic fibroblasts (MEFs) but not MK2-null MEFs (Fig. 2d). Additionally, only constitutively active MK2, but not kinase-dead MK2 augmented AP1 activity in MK2-null MEFs and ER + MCF7 cells (Fig. 2e). These results suggest that p38^MAPK^-MK2 signaling pathway contributes to elevated AP1 activity in TNBC cells.

### JAB1 interacts with MK2 and its presence contributes to robust AP1 activity in TNBC cells

**Fig. 3:**
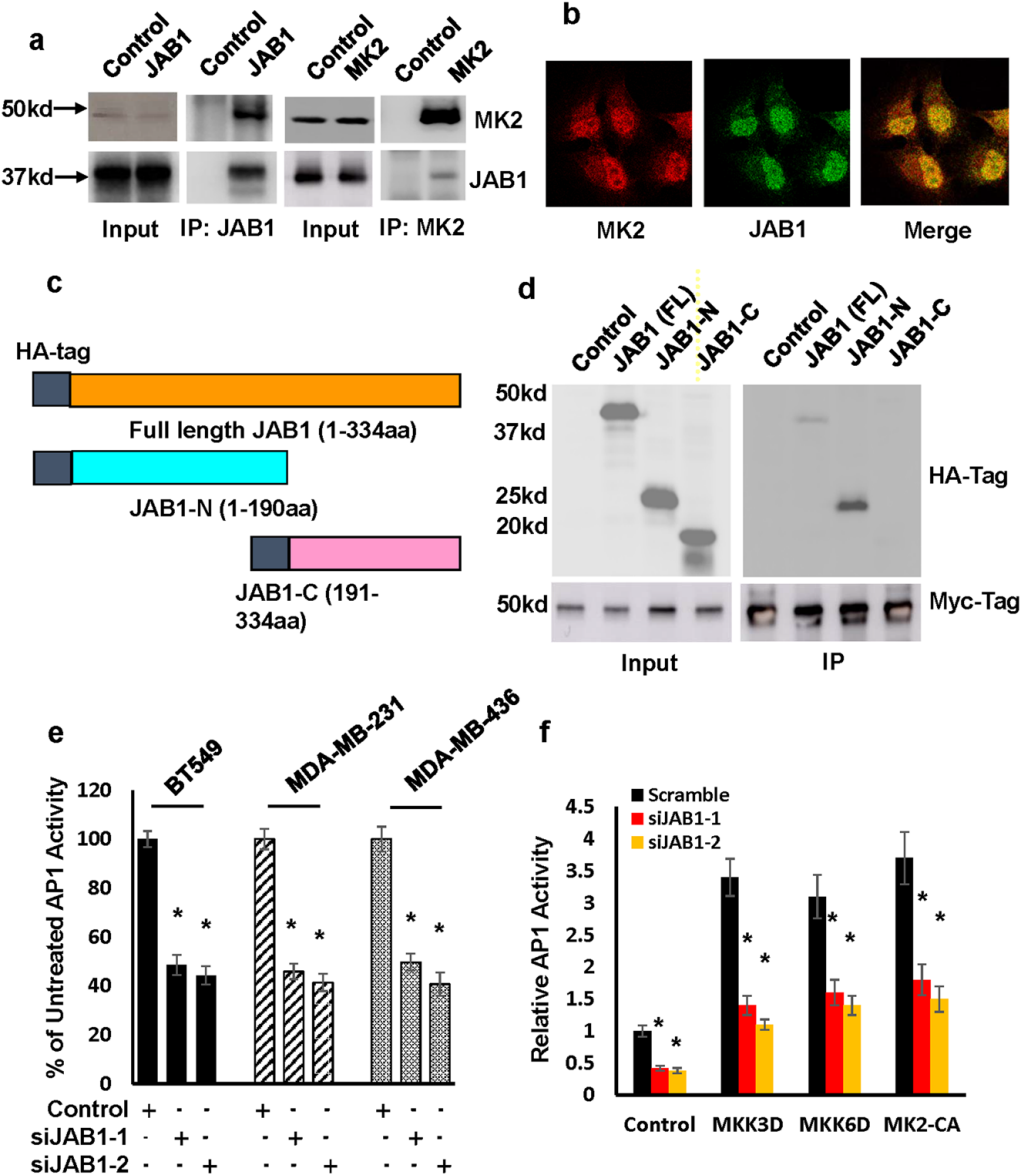
JAB1 is an MK2 substrate and is required for robust AP1 activity in TNBC cells. **a** MDA-MB-231 cells were immunoprecipitated with either JAB1 or MK2 antibody and the immunoprecipitates were subjected to western blot to detect MK2 or JAB1 respectively. All blots derived from the same experiment and were processed in parallel. **b** MDA-MB-231 cells were subjected to immunofluorescence staining to detect JAB1 and MK2. Data are the representative of four independent experiments. **c** Diagram of HA-tagged full-length JAB1, N-terminal Jab1 (JAB1-N), and C-terminal JAB1 (JAB1-C). **d** HEK293 cells were transfected with empty plasmid (Control) or with plasmids encoding HA-tagged full-length JAB1 (FL), JAB1-N or JAB1-C, and cell lysates were immunoprecipitated with MK2 pAb. Immunoprecipitates were subjected to western blot analysis to detect interactions between the JAB1 proteins and MK2 by HA mAb. Data are the representative of two independent experiments. All blots derive from the same experiment and were processed in parallel. **e** BT549, MDA-MB-231 or MDA-MB-436 cells were treated with 30 nM JAB1 siRNA (siJAB1-1 and siJAB1-2) or scrambled siRNA control for 3 days followed by analyzing AP 1 activity. Data are means ± SD from three experiments. *P < 0.01 vs Control. **f** Wild-type MEFs were first transduced with MKK3D, MKK6D, MK2-CA, or empty vector (Control) for 3 days and then treated with 30 nM JAB1 siRNAs for 3 days followed by analyzing AP1 activity. Data are means ± SD from three experiments. *P < 0.01 vs Control.

To elucidate molecular mechanism underlying MK2 regulation of AP1 activity, we analyzed the results on our previously performed yeast two-hybrid screening and noticed JAB1 as one of the MK2 binding partners^26^. JAB1 is a known JUN-binding protein and enhances AP1 activity by stabilizing AP1 binding to AP1 sites^19^, which raised the possibility that p38^MAPK^-MK2 signaling pathway regulates AP1 activity in a mechanism that encompasses JAB1. To test this possibility, we experimentally verified JAB1 and MK2 interaction by co-immunoprecipitation experiments in MDA-MB-231 cells using either antibody against MK2 or antibody against JAB1. Western blot analysis showed that JAB1 was present in the MK2 immunoprecipitates while MK2 in JAB1 immunoprecipitates (Fig. 3a). Subsequent confocal microscope-based immunofluorescence staining further revealed the co-localization of JAB1 and MK2 in MDA-MB-231 cells (Fig. 3b).

To determine whether the activation status of MK2 affected JAB1 and MK2 interaction, we transfected HEK293 cells with plasmids encoding Myc-tagged wild-type MK2, constitutively active MK2 (MK2-CA) or dominant negative MK2 (MK2-DN) followed by co-immunoprecipitation with an antibody against JAB1. Immunoblotting showed that MK2, MK2-CA, and MK2-DN all interacted with JAB1 (Supplementary Fig. 2). To map the region in JAB1 that was required for its interaction with MK2, we transfected HEK293 cells with plasmid encoding the hemagglutinin (HA)-tagged full-length, N- or C-terminal regions of JAB1 along with a plasmid encoding Myc-tagged MK2 (Fig. 3c). Co-immunoprecipitation experiments showed that only N-terminal region of JAB1 interacts with MK2 (Fig. 3d).

We next assessed the effect of silencing JAB1 on AP1 activity in TNBC cells by lentivirally introducing JAB1 shRNAs into BT549, MDA-MB-231 and MDA-MB-436 cells. Luciferase-aided AP1 reporter assay showed that both JAB1 shRNAs were able to diminish over 50% of AP1 activity (Fig. 3e). In a parallel experiment, we found that JAB1 knockdown also abolished the ability of constitutively active MKK3, MKK6, or MK2 to enhance AP1 activity in MEFs (Fig. 3f). These results provide clear evidence that the presence of JAB1 is essential for p38^MAPK^-MK2 signaling pathway regulation of AP1 activity.

### JAB1 is a direct substrate of MK2 and MK2-mediated JAB1 phosphorylation is required for p38^MAPK^-MK2 regulation of AP1 activity

**Fig. 4:**
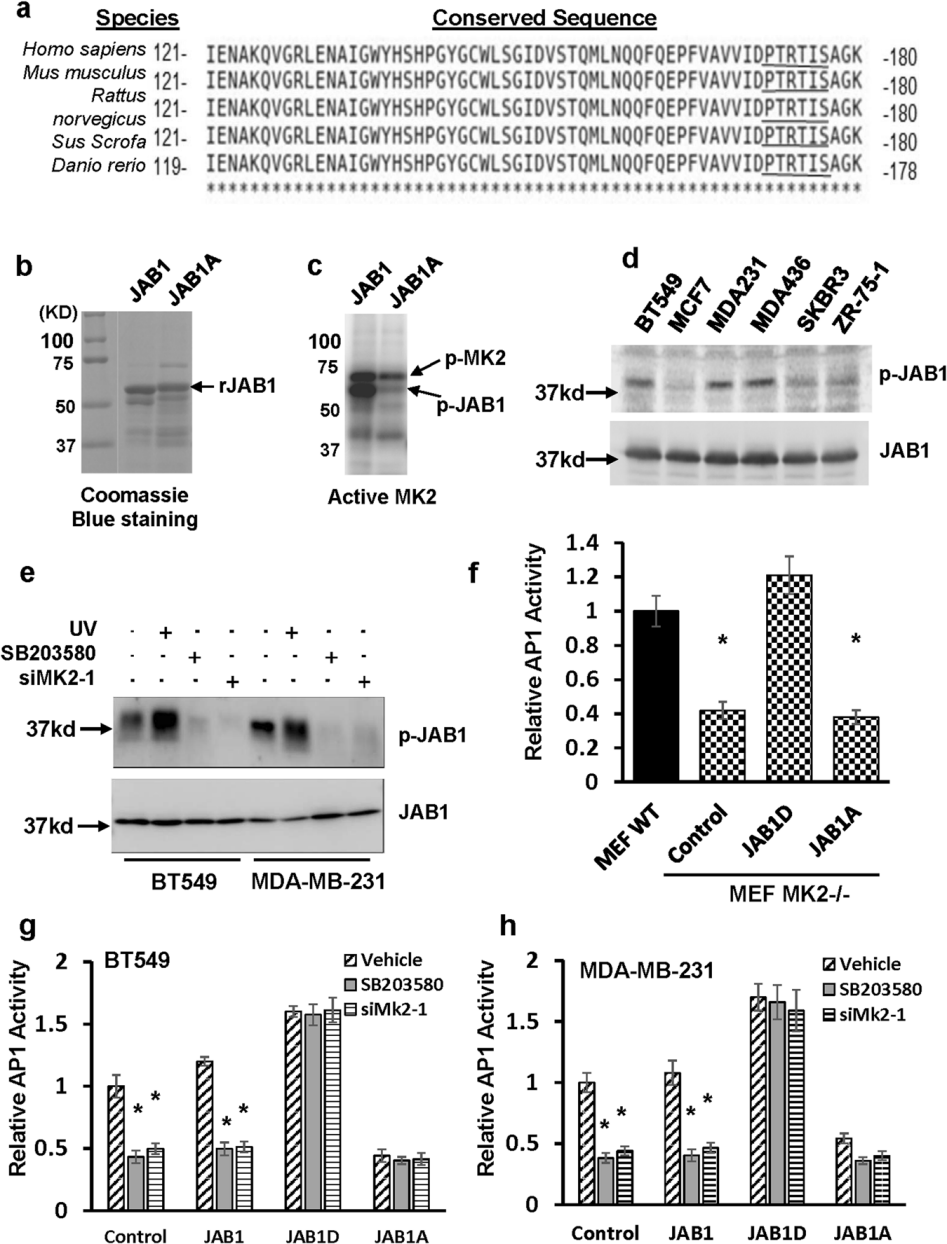
MK2 phosphorylates JAB1 at Ser177 and Ser177 of JAB1 is essential for robust AP1 activity in TNBC cells. **a** Alignment of JAB1 protein sequences from different species. The conserved MK2 phosphorylation target motif is underlined. Amino acid residue numbers are to the left and right of the sequences. **b** Coomassie blue staining of recombinant JAB1 and JAB1A. **c** In vitro kinase assay with recombinant active MK2 to determine the ability of MK2 to phosphorylate JAB1. Data are the representative of two independent experiments. **d** Western blot analysis to detect Ser^177^-phosphorylated JAB1 (p-JAB1) in breast cancer cell lines. **e** BT549 or MDA-MB-231 cells were under UV exposure for 1 min, treated with 5 µM SB203580 for 1 days, 30 nM MK2 siRNA (siMK2-1) for 3 days or left untreated followed by western blot analysis to detect Ser^177^-phosphorylated JAB1 and JAB1. Data are the representative of two independent experiments. All blots derived from the same experiment and were processed in parallel. **f** MK2−/− MEFs were transduced with JAB1D, JAB1A or empty vector for 3 days followed by analyzing AP1 activity. Wild-type MEF was included for comparison. Data are means ± SD from three experiments. *P < 0.01 vs wild-type MEF (MEF WT). **g**, **h** BT549 (**g**) and MDA-MB-231 cells (**h**) were first transduced with JAB1, JAB1D, JAB1A, or empty vector (Control) for 3 days and then treated with 5 µM SB203580, vehicle, or 30 nM MK2 siRNA for 3 days followed by analyzing AP1 activity. Data are means ± SD from three experiments. *P < 0.01 vs Vehicle.

MK2 is a serine kinase and its consensus phosphorylation motif is Hyd-X-Arg-X-X-Ser, where Hyd is a hydrophobic residue. Search of the JAB1 amino acid sequence revealed a corresponding sequence PTRTIS^177^ at amino acid residues 172–177, and this sequence is conserved across different species (Fig. 4a). To determine whether this site is a true MK2 phosphorylation site on JAB1, we generated recombinant wild-type JAB1 and mutant JAB1A (JAB1-S177A) in which Ser^177^ was mutated to Ala (Fig. 4b). In vitro kinase assay with recombinant active MK2 showed that recombinant JAB1 protein was robustly phosphorylated while phosphorylation of JAB1-S117A was negligible (Fig. 4b). Moreover, we performed Mass Spectrometry to determine the phosphorylation site(s) in MK2-phosphorylated recombinant JAB1 and confirmed that Ser^177^ of JAB1 was indeed phosphorylated (Supplementary Fig. 3). Taken together, these results show that MK2 can directly phosphorylate JAB1 at Ser^177^.

To determine whether MK2 also phosphorylates JAB1 at Ser^177^ in TNBC cells, we generated a polyclonal antibody specific for phosphorylated JAB1 with a synthetic Ser^177^-phosphorylated peptide [AVVIDPTRTI(pS)AGKVN], which corresponded to amino acid residues of human JAB1 protein. Western blot analysis showed that this antibody was able to detect recombinant JAB1, but not JAB1-S177A that were pre-incubated with recombinant active MK2 (Supplementary Fig. 4a). Parallel western blot with this antibody also showed that level of phosphorylated JAB1 increased in MCF7 cells treated with UV or anisomycin, two known activators of p38^MAPK^-MK2 signaling pathway over the untreated cells (Supplementary Fig. 4b). In contrast, western blot with anti-JAB1 antibody did not exhibit any alteration in the abundance of JAB1 between treated and untreated cells (Supplementary Fig. 4b). These results thus establishes the specificity of this antibody to detect Ser^177^-phosphorylated JAB1. Consistent with JAB1 as a MK2 substrate, western blot analysis showed that TNBC lines (BT549, MDA-MB-231 and MDA-MB-436), which exhibited higher p38^MAPK^ and MK2 activities (Figs. 1 and 2), displayed higher level of Ser^177^-phosphorylated JAB1 than ER + lines (Fig. 4d). Exposing BT549, MDA-MB-231 and MDA-MB-436 cells to UV further increased the level of Ser^177^-phosphorylated JAB1 while SB203580 treatment or knockdown of MK2 reduced the amount of Ser^177^-phosphorylated JAB1 (Fig. 4e and Supplementary Fig. 5). Taken together, these results raised the possibility that MK2-mediated JAB1 Ser^177^ phosphorylation could be functionally associated with MK2 regulation of AP1 activity in TNBC cells.

Since the presence of JAB1 is necessary for MK2-led induction in AP1 activity and is a direct substrate of MK2, we went on to investigate whether MK2-mediated JAB1 phosphorylation was relevant to AP1 activity. We lentivirally introduced wild-type JAB1, an MK2-phosphomimetic mutant form of JAB termed as JAB1D (in which Ser^177^ is replaced by Asp) or an MK2-nonphosphorylatable mutant form of JAB1 termed as JAB1A (in which Ser^177^ was replaced by Ala) into MK2-null MEFs. Luciferase-aided AP1 reporter assay showed that AP1 activity was only increased by JAB1D (Fig. 4f). In parallel, we ectopically expressed JAB1A or JAB1D into BT549 and MDA-MB-231 cells followed by either SB203580 treatment or MK2 knockdown. JAB1 D, but not JAB1A, restored the AP1 activity in SB203580-treated or MK2-knockdown cells (Fig. 4g, h). These results are consistent with the notion that MK2-mediated Ser^177^ phosphorylation of JAB1 is important for p38^MAPK^-MK2 regulation of AP1 activity.

### The presence of JAB1 is critical for AP1-mediated cyclin D1, uPA and uPAR expression in TNBC cells

**Fig. 5:**
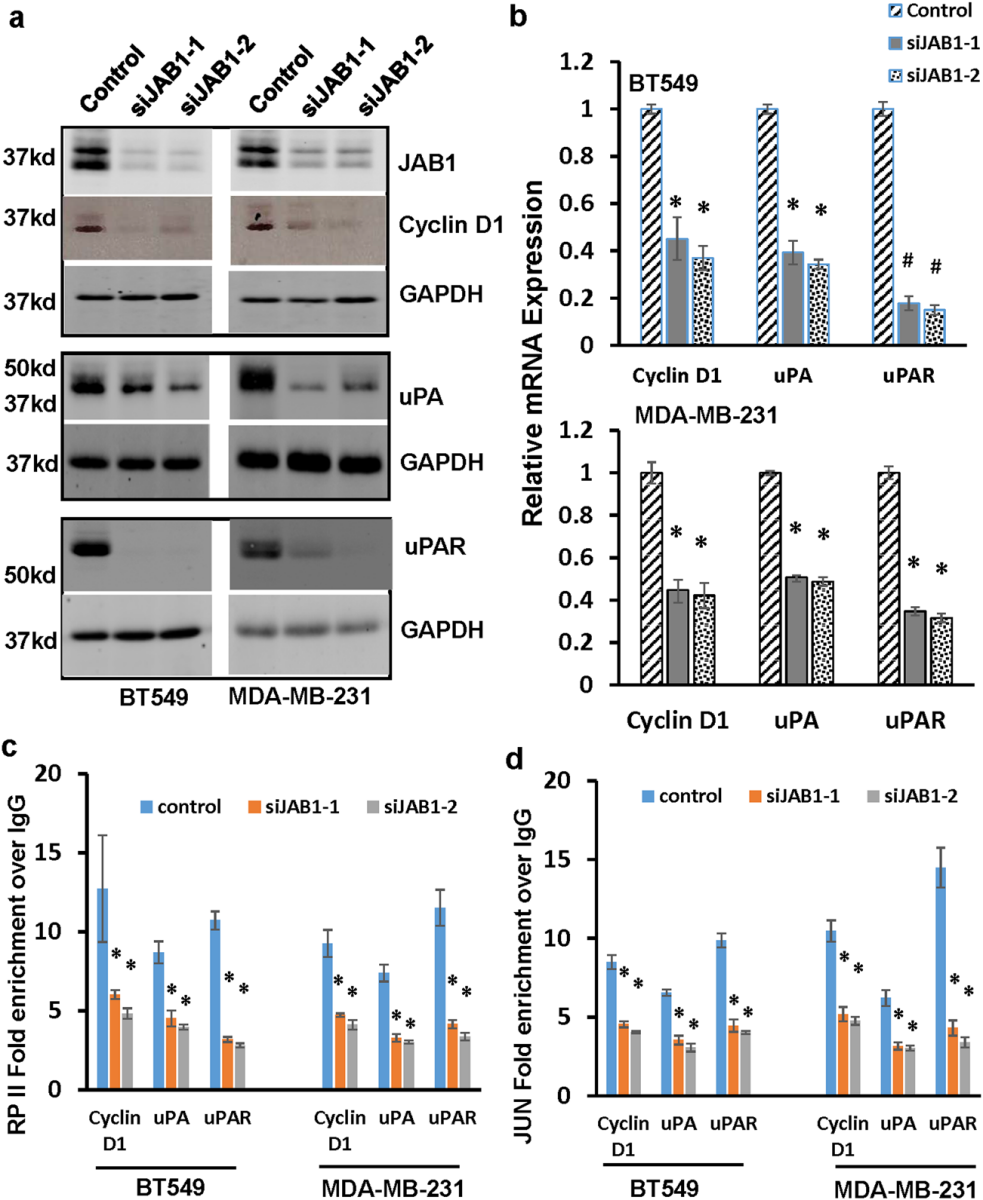
The presence of JAB1 is required for AP1-driven transcription in TNBC cells. **a** BT549 or MDA-MB-231 cells were treated with 30 nM JAB1 siRNAs (siJAB1-1, siJAB1-2) or scrambled siRNA control for 3 days followed by western blot analysis to detect JAB1, cyclin D1, uPA, uPAR, and GAPDH with the respective antibodies. Data are the representative of two independent experiments. All blots derived from the same experiment and were processed in parallel. **b** BT549 or MDA-MB-231 cells were treated with 30 nM JAB1 siRNAs or scrambled siRNA control for 3 days followed by qRT-PCR to measure the levels of Cyclin D1, uPA and uPAR mRNA. Level of βActin mRNA was used for standardization. Data are means ± SD from three experiments. *P < 0.01 vs Control; #*P* < 0.005 vs Control. **c**, **d** BT549 or MDA-MB-231 cells were treated with 30 nM JAB1 siRNAs or scrambled siRNA control for 3 days and then subjected to RP-II (**c**) or JUN ChIP (**d**) followed by qPCR to analyze RP-II or JUN occupancy in cyclin D1, uPA and uPAR promoters. Data are means ± SD from three experiments. * *P* < 0.01 vs Control.

The ability of JAB1 to modulate AP1 activity prompted us to investigate how knockdown of JAB1 affected the expression of cyclin D1, uPA and uPAR which are known to be regulated in AP1-dependent manner^27–29^. Western blot analysis showed that silencing JAB1 led to marked reduction in the abundance of all these three proteins in both BT549 and MDA-MB-231 cells (Fig. 5a). QRT-PCR revealed similar levels of reduction in mRNAs of these three (Fig. 5b). The reduced expression was apparently caused by less transcription because chromatin immunoprecipitation (ChIP) with RNA polymerase II (RP-II) pAb showed that knockdown of JAB1 diminished over 50% of RP-II occupancy in their respective promoter sequences (Fig. 5c). In a parallel experiment, we performed ChIP with JUN mAb followed by qPCR with primers specific for previously characterized AP1 binding sites in cyclin D1, uPA and uPAR promoters^28–30^. QPCR showed that sequences of AP1-containing regions in these promoters were enriched at least 5 fold in JUN immunoprecipitates over the IgG control (Fig. 5d). However, such enrichment was ~40–60% less in JAB1-knockdown cells (Fig. 5d). These results suggest that the presence of JAB1 is critical for robust AP1-driven transcription in TNBC cells.

To elucidate molecular mechanism underlying JAB1’s promoting role in AP1 activity, we initially determined whether the presence of JAB1 or its Ser177 phosphorylation status affected c-JUN abundance. Western blot analysis showed that neither silencing JAB1 nor forced expression of JAB1A (non-MK2-phosphorable) altered the amount of JUN in MDA-MB-231 cells (Supplementary Fig. 6). We next examined whether the presence of JAB1 is essential for either homo- or hetero-dimerization of JUN. Co-immunoprecipitation of MDA-MB-231 cell lysates showed the presence of JUN homo-dimerization or hetero-dimerization of JUN with Fra-1 and ATF2 (Supplementary Figs. 7 and 8a, b). However, such dimerization did not appear to be affected by the knockdown of JAB1 (Supplementary Figs. 7 and 8a, b). We lastly investigated whether Ser^177^ phosphorylation status of JAB1 affected its interaction with JUN. MDA-MB-231 cells were treated with SB203580 to inhibit p38^MAPk^ activity followed by immunoprecipitation with anti-JUN or anti-JAB1 polyclonal antibody. Western blot analysis of theses immunoprecipitates showed that SB203580 had little effect on the amount of JAB1 in JUN immunoprecipitates (Supplementary Fig. 9a) or JUN in JAB1 immunoprecipitates (Supplementary Fig. 9b). In a parallel experiment, we performed immunoprecipitation in MDA-MB-231 cells transduced with HA-tagged JAB1, JAB1D, or JAB1A using HA mAb. Western blot analysis of these immunoprecipitates with anti-JUN polyclonal antibody showed that c-JUN was able to indiscriminately interact with all three JAB1 forms (Supplementary Fig. 10), indicating that MK2-mediated JAB1 phosphorylation is unlikely to regulate JUN-driven AP1 activity by controlling JAB1-JUN interaction.

### MK2-mediated JAB1 phosphorylation facilitates JUN recruitment to AP1 consensus sequences

**Fig. 6:**
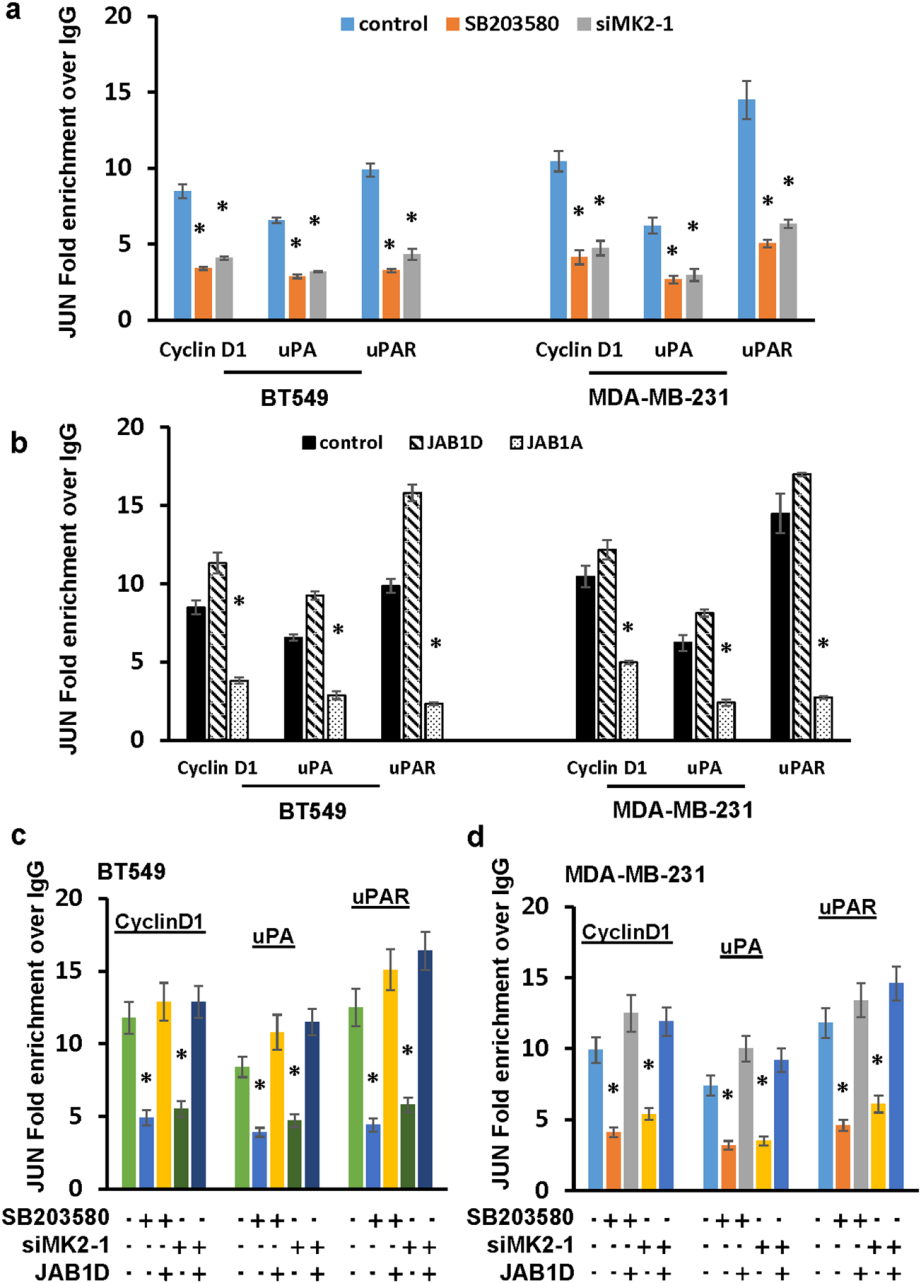
Ser177 phosphorylation of JAB1 impacts JUN binding to AP1 sites. **a** BT549 or MDA-MB-231 cells were treated with 5 µM SB203580 for 1 day or 30 nM MK2 siRNA (siMK2-1) for 3 days for left untreated (control) and then subjected to JUN ChIP followed by qPCR to analyze JUN occupancy in cyclin D1, uPA and uPAR promoters. Data are means ± SD from three experiments. *P < 0.01 vs Control. **b** BT549 or MDA-MB-231 cells were transduced with JAB1D, JAB1A, or empty vector (control) for 3 days and then subjected to JUN ChIP followed by qPCR to analyze JUN occupancy in cyclin D1, uPA and uPAR promoters. Data are means ± SD from three experiments. *P < 0.01 vs Control. **c**, **d** BT549 (**c**) or MDA-MB-231 cells (**d**) were transduced JAB1D or empty vector for 3 days and then treated with 5 µM SB203580 for 1 day or 30 nM MK2 siRNA (siMK2-1) for 3 days followed by JUN ChIP to detect JUN occupancy in cyclin D1, uPA and uPAR promoters. Data are means ± SD from three experiments. *P < 0.01 vs Control.

To functionally link Ser^177^ phosphorylation of JAB1 to JUN-driven transcription, we initially investigated the involvement of p38^MAPK^-MK2 signaling on JUN recruitment to cyclin D1, uPA and uPAR promoter by performing JUN ChIP in BT549 and MDA-MB-231 cells treated with SB203580 or with MK2 knockdown. Inhibition of p38^MAPK^ activity or silencing MK2 led to 50–60% reduction in JUN occupancy of the AP1 binding sites in these promoters (Fig. 6a). These results raised the possibility that Ser^177^ phosphorylation of JAB1 impacts JUN binding to AP1 sites. To test this possibility, BT549 and MDA-MB-231 cells with the forced expression of JAB1D or JAB1A were subjected to ChIP with JUN mAb. QPCR showed that ectopic JAB1D expression further increased JUN occupancy in cyclin D1, uPA and uPAR promoters (Fig. 6b). In contrast, JAB1A reduced ~50% JUN recruitment to these promoters (Fig. 6b). Moreover, JAB1D restored JUN occupancy to these promoters in BT549 and MDA-MB-231 cells treated with SB203580 or depleted of MK2 (Fig. 6c, d). These results suggest that Ser^177^ phosphorylation of JAB1 positively regulates AP1 activity by facilitating JUN binding to AP1 sites.

### JAB1 play a critical role in breast cancer tumorigenesis

**Fig. 7:**
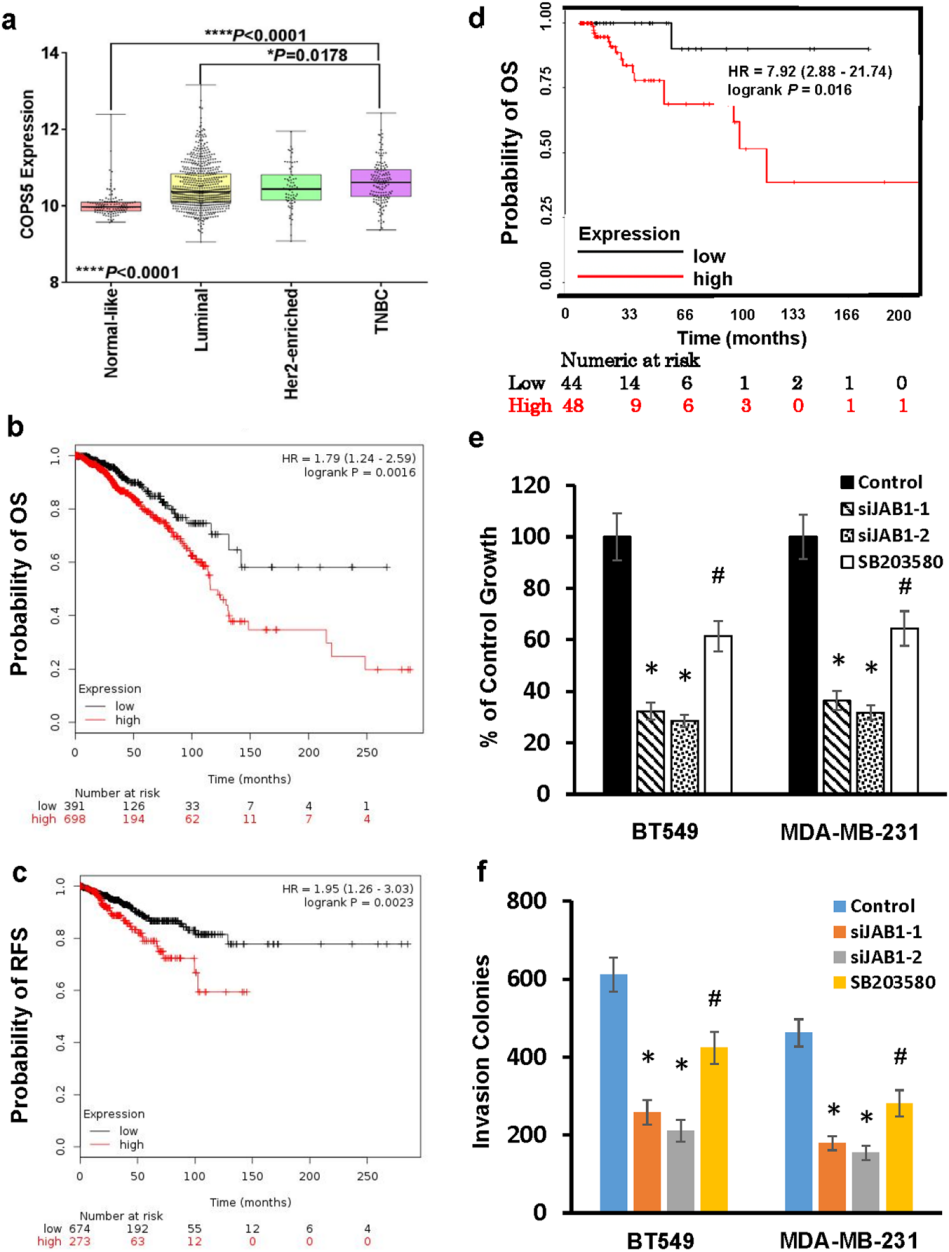
JAB1 is functionally associated with TNBC tumorigenesis. **a** Box-and-whisker plot was generated to show the expression of JAB1 in breast tumor subtypes. Statistical analysis was performed using GraphPad Prime 7.0 with unpaired *t* test (two-tailed). **b**, **c** JAB1 expression is inversely correlated with breast cancer patient overall (**b**) and recurrence-free survival (**c**). **d** JAB1 expression is inversely correlated with TNBC patient overall survival. **e**, **f** BT549 or MDA-MB-231 cells were treated with 30 nM JAB1 siRNAs (siJAB1-1, siJAB1-2), scrambled siRNA control for 3 days or 5 µM SB203580 for 1 day followed by MTT assay to analyze cell growth (**e**) or Matrigel invasion assay to determine in vitro invasion (**f**). Data are means ± SD from three experiments. *P < 0.01 vs Control. #*P* < 0.05.

The essence of AP1 transcription activity in cancer development and progression prompted us to investigate the tumorigenic role of JAB1. We first assessed the correlation between JAB1 expression and clinical features of breast cancer patients by analyzing TCGA breast tumor dataset. High JAB1 expression correlated with positive lymph node status, pathological stage and tumor recurrence (*p* = 0.004, 0.038, and 0.041 respectively; Supplementary Table 1). Further analysis of its expression in various breast cancer subtypes revealed that JAB1 expression is higher in TNBCs over the other subtypes (Fig. 7a). Univariate survival analysis (Kaplan–Meier method, log-rank test) showed that high JAB1 expression was associated with both short overall and recurrence-free survival rate of patients (Fig. 7b, c). When only data from TNBC patients were analyzed, we found that high JAB1 expression was again associated with poor overall survival (Fig. 7d). These findings support the notion that JAB1 is involved in breast tumorigenesis.

To elucidate contribution of JAB1 toward TNBC tumorigenic events, we assessed the effect of silencing JAB1 on TNBC cell growth, migration, and in vitro invasion. Knockdown of JAB1 led to the reduced growth and in vitro invasion in both BT549 and MDA-MB-231 cells (Fig. 7e, f), consistent with the observation that levels of cyclin D1, uPA and uPAR were downregulated in cell with JAB1 knockdown. Interestingly, JAB1 knockdown led to greater inhibition in both cell growth and invasion than SB203580 did, indicating that JAB1 is involved in TNBC tumorigenesis more than just participating in p38^MAPK^-MK2 signaling pathway.

**Fig. 8:**
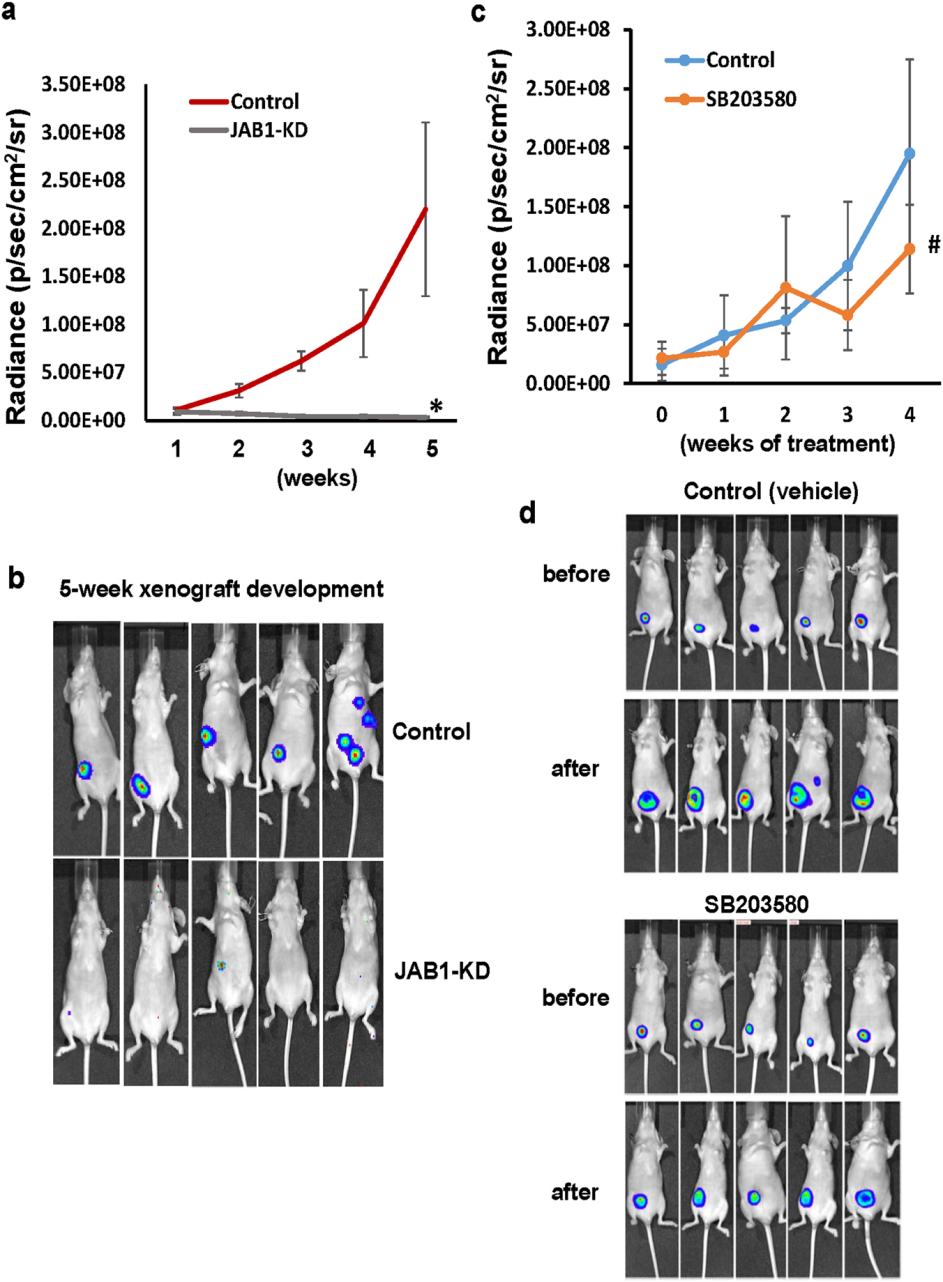
The presence of JAB1 is critical for tumor development. **a** Luciferase-expressing MDA-MB-231 cells were transduced with lentiviral vector encoding either scramble or JAB1 shRNA for 3 days and then subcutaneously injected into nude mice. Tumor outgrowth was monitored weekly using the Xenogen IVIS-200 In Vivo bioluminescence imaging system. Data are means ± SD. *n* = 5. Statistical analysis was performed using Student *t* test (two-tailed) for the end experiment point. *P < 0.001. **b** Images of the xenograft tumors at 5 week. The image data is displayed in radiance or photons/sec/cm^2^/steradian. **c** Luciferase-expressing MDA-MB-231 cells were injected into nude mice for 5 days. Mice were randomized and treated daily with either Vehicle control or 1 µmol/kg of SB203580 i.p. for 4 weeks. Tumor growth were monitored weekly though Xenogen IVIS-200 in vivo imaging system. Data are means ± SD. *n* = 5. Statistical analysis was performed using Student’s *t* test (two-tailed) for the end experiment point. #*P* < 0.05. **d** Images of the xenograft tumors before treatment and after 4 weeks of treatment.

In subsequent experiments, we analyzed the effect of JAB1-knockdown in TNBC tumor development. Luciferase-expressing MDA-MB-231 cells were lentivirally transduced with control (expressing scramble shRNA) or JAB1 shRNA and then injected into mammary fat pad area of female athymic nude mice. Bioluminescence imaging showed that control cells rapidly progressed (Fig. 8a, b). In contrast, JAB1-knockdown cells barely formed tumors (Fig. 8a, b), indicating that in vivo tumor development depends on the presence of JAB1. In a parallel experiment, we investigated the essence of p38^MAPK^ activity on tumor outgrowth. Luciferase-expressing MDA-MB-231 cells were injected to mice followed by daily administration of vehicle or SB203580. Similar to what we observed with tumorigenic events, SB203580 deterred tumor outgrowth compared to mice receiving only vehicle, though its suppressing effect was far less pronounced than JAB1 knockdown (Fig. 8c, d).

## Discussion

AP1 activity has long been recognized for its role in tumorigenesis^1,2^. For example, JUN-containing AP1 complex induces the expression of positive regulators of cell cycle such as cyclin D1 while represses negative regulators such as p53 and INK4A^31^. Fra-1 can facilitate cancer cell invasiveness by transcriptionally upregulating the expression of wide range of proteases including uPA and metalloproteinases^32,33^. Consistent with its role in cancer cell invasiveness, we noticed AP1 activity is much higher in invasive TNBC cells than non-invasive ER + breast cancer cells (Fig. 1). Although MAPKs including ERK, JNK and p38 have been shown to regulate AP1 activity through phosphorylation of AP1 components in various cell types^2^, their role in AP1 activity of breast cancer remains elusive. To establish functional link between AP1 and MAPK signaling pathways, we show that robust AP1 activity in TNBC cells involves both ERK and p38^MAPK^, but not JNK signaling pathways (Fig. 1). This is apparently in accordance with our previous findings^15^ and the observations that the abundance of phosphorylated ERK and p38^MAPK^ is much higher in TNBC cells than ER + breast cancer cells (Fig. 2). The importance of ERK signaling pathway can be readily explained as the expression of JUN depends on activity of ERK signaling pathway (Fig. 1). In a previous report, p38^MAPK^ was found to mediate UV-induced AP1 activation by directly phosphorylating FOS^17^. However, FOS is hardly detectable in TNBC cells^15^ and we did not notice apparent alteration in the abundance of JUN upon SB203580 treatment (Fig. 1), indicating that a previously unidentified mechanism is responsible for p38^MAPK^ regulation of AP1 in TNBC cells. In fact, we show that p38^MAPK^ signaling pathway promotes AP1 activity through MK2, a well identified downstream effector of p38 MAPK (Fig. 2).

MK2 is well identified for its role in inflammatory responses and serves as a master regulator of RNA binding proteins for modulating cytokine mRNA stability^34,35^. In addition, MK2 has also been linked to tumor progression and metastasis. For example, MK2 regulates actin polymerization and remodeling by directly phosphorylating Hsp27^36^, which is crucial for cancer cell migration and invasion^37,38^. MK2 can also promote cell survival in cells under DNA damage by dampening the p53-mediated response to DNA damage/stress^39,40^. Here, we found that knockdown of MK2 led to significant reduction in AP1 activity in TNBC cells and that only constitutively active MK2 was able to increase AP1 activity in MK2-null MEF cells (Fig. 2). Given the well characterized role of AP1 in tumorigenesis, we uncovered a previously unknown mechanism underlying MK2 regulation of cancer progression.

In an effort to understand MK2 action, we identified JAB1 as an MK2-interacting protein (Fig. 3). Importantly, knockdown of JAB1 led to reduction in endogenous AP1 activity in TNBC cells (Fig. 3). The importance of JAB1 for AP1 activity is likely associated with p38^MAPK^ signaling pathway because the depletion of JAB1 blocked the enhanced AP1 activity conferred by constitutively active MKK3/6, upstream kinases of p38^MAPK^, and MK2 (Fig. 3). JAB1 was initially identified as a JUN-binding protein capable of increasing AP1 activity though the stabilization of AP1-AP1 site interaction^19^. This is apparently in agreement with our observation that silencing JAB1 resulted in >50% reduction in JUN enrichment in AP1 sites of cyclin D1, uPA and uPAR promoters (Fig. 5). Taken together, we reason that p38^MAPK^ signaling pathway facilitates AP1 activity by stabilizing AP1 complex binding to AP1 sites through JAB1 in TNBC cells. The nature of MK2 as a kinase indicates that MK2 may exert its function by directly phosphorylating JAB1. In fact, we show that MK2 phosphorylates JAB1 at Ser^177^ both in vitro and in TNBC cells (Fig. 4). This MK2-mediated phosphorylation is critical for JAB1’s ability to augment AP1 activity because non-MK2-phosphorylable (JAB1-S177A) was unable to increase AP1 activity while MK2 phosphor mimetic of JAB1 (JAB1-S177D) increased AP1 activity even in the presence of SB203580 or upon MK2 depletion (Fig. 4). Recently, MK2 was reported to control TNF-associated cytotoxic signaling by phosphorylating RIPK1^41–43^. We previously showed that MK2 promotes efficient miRNA biogenesis by controlling subcellular localization of DDX5 through direct phosphorylation^26^. This study presents another example that MK2 regulates the function of its downstream molecule through direct phosphorylation.

Consistent with JAB1’s role in promoting AP1 activity, we show that knockdown of JAB1 led to over 50% reduction in enrichment of JUN in AP1 sites of cyclin D1, uPA and uPAR promoters and levels of respective mRNAs (Fig. 5). Since JUN-AP1 site interaction is decreased by SB203580, depletion of MK2 or non-MK2-phosphorylable JAB1 mutant (Fig. 6), it raises the possibility that AP1 activity is regulated by the Ser^177^ phosphorylation of JAB1. This notion is clearly supported by the observation that Ser^177^ phosphor mimetic of JAB1 restored JUN-AP1 site interaction in TNBC cells either treated with SB203580 or depleted with MK2 (Fig. 6). MK2-mediated phosphorylation has been found to regulate subcellular localization of its substrate including DDX5^26^, tristetraprolin^44,45^, and HuR^46,47^. It can also control protein–protein interaction as phosphorylation of PDE4A5 by MK2 enhances PDE4A5 interaction with p75NTR^48^. However, we noticed that JAB1-S177D and JAB1-S177A displayed similar extent of its interaction with JUN (Supplementary Fig. 8). As JUN-JAB1 interaction is expected to be present in nucleus, these observations also indicate that neither subcellular localization of JAB1 nor JAB1-JUN interaction was affected by MK2-mediated phosphorylation. Moreover, we found that MK2-mediated phosphorylation did not affect JUN abundance (Supplementary Fig. 4) and the formation of JUN-containing AP1 complexes (JUN-JUN, JUN-Fra-1, and JUN-ATF2 dimers) (Supplementary Figs. 5, 6, and 7). Instead, we found that MK2-mediated phosphorylation of JAB1 impacted the degree of JUN enrichment to AP1 sites (Fig. 6). These results suggest that Ser^177^ phosphorylation of JAB1 facilitates the binding of JUN-containing AP1 complex to AP1 sites, which probably is similar to a previous study in which phosphorylation of β-catenin by AKT was found to promote β-catenin-containing TCF/LEF transcription activity^49^.

JAB1 has been linked to tumorigenesis of various cancer types including breast^50,51^, pancreatic^52^, lung^53^, and liver cancer^54^. Analysis of TCGA dataset revealed that its expression is correlated with both shorter overall and recurrence-free survival in breast cancer patients (Fig. 7). Consistent to its tumorigenic role, we observed that knockdown of JAB1 diminished TNBC cell growth, in vitro invasion and tumor outgrowth (Figs. 7 and 8). Intriguingly, tumor-suppressive effect elicited by JAB1 knockdown is >p38^MAPK^ inhibitor SB203580 (Figs. 7 and 8), indicating that JAB1 regulation of TNBC tumorigenesis may be multi-pronged and one prong being the p38^MAPK^/MK2 signaling pathway. In fact, JAB1 has been found to facilitate the degradation of tumor-inhibitory proteins such as p53^22^, p27^24^, p57^55^, Bax, and Smad4^25^. Especially, JAB1/S100A7 can enhance prosurvival pathway in breast cancer cells^21^. Taken together, our results suggest that enhancing AP1 activity is most likely one of the mechanisms pertinent to tumor-promoting role of JAB1 in TNBC.

In summary, we show that both ERK and p38^MAPK^ signaling pathways are involved in sustaining robust AP1 activity in TNBC cells and further provide detailed molecular mechanisms underlying p38^MAPK^ signaling pathway regulation of AP1 activity. Our data suggest that MK2/JAB1 is the mechanism linking p38^MAPK^ signaling pathway to AP1 activity.

## Methods

### Cell lines, siRNAs, and shRNA-containing lentiviral vectors

BT549, MCF-7, MDA-MB-231, MDA-MB-436, SKBR3, and ZR-75-1 cell lines were obtained from ATCC (Manassas, VA) and maintained in Dulbecco’s modified Eagle’s high glucose medium containing 10% fetal bovine serum (Hyclone, Logan, UT) in a humidified incubator at 37 °C and 5% CO2. Jab siRNAs (Catalog # MQ-005814-01) and MK2 siRNAs (Catalog # MQ-003516-02) siGENOME siRNA were purchased from Horizon Discovery (Cambridge, UK) shRNAs oligonucleotides for JAB1 gene were designed with the aid of web-based Invitrogen Block-It program. The prepared shRNA oligonucleotides were inserted into GFP-containing pLV-shRNA vector (Biosettia, San Diego, CA) according to manufacturer’s protocol. The sequence of JAB1 shRNA for generating JAB1-knockdown cells is 5′-gcttgagctgttgtggaataa-3′.

### Cell growth and in vitro invasion assay

Cell growth was analyzed by MTT assay as described previously^26^. Briefly, cells were cultured in 24-well plates overnight and then treated with 5 μM SB203580 for 3 days followed by incubation with MTT solution to a working concentration of 5 μg/ml for 2 h in humidified incubator with 5% CO^2^ at 37 °C. After that media were removed and MTT formazan crystals were dissolved in DMSO and measured with a Bio-Rad plate reader at a wavelength of 560 nm. Similarly, cells were treated with 30 nM JAB1 siRNAs (siJAB1-1, siJAB1-2), scrambled siRNA control for 3 days and then subjected to MTT assay as described above. In vitro cell invasion was assayed using Matrigel invasion chamber available from Cell Biolabs Inc (San Diego, CA) according to manufacturer’s protocol.

### AP1 activity assay

AP1 transcriptional activity was measured with the aid of a firefly luciferase reporter gene plasmid (3xAP1pGL3) from Addgene (Watertown, MA). This plasmid contains three copies of AP1 consensus sequence and was previously used to monitor AP1 activity in B cells^56^. To determine cellular AP1 activity, this plasmid was co-transfected into cells with a Renilla luciferase gene-containing plasmid (pRL-TK, Promega, Madison, WI) using lipofectamin 2000 (Invitrogen, Carlsbad, CA). After 36 h, cells were lysed and cell lysates were assayed for luciferase activity using Dual-Luciferase® Reporter Assay System (Promega Corp, Madison, WI). Renella luciferase activity was used as an internal transfection control for standardization.

### Immunofluorescent staining

Cells were plated on coverslips for overnight and then fixed with 4% paraformaldehyde followed by permeabilization with 1% Triton X-100. After brief blocking with 1% BSA and 10% serum, cells were incubated with primary antibodies anti-Jab1 mAb (MA1-23244 at 5 μg/ml dilution, Thermo Scientific, Waltham, MA) or anti-MK2 pAb (sc-7871 at 1:200 dilution, Santa Cruz Biotechnology, Santa Cruz, CA) for 1 h and then with secondary antibody (Alexa Fluor 488 goat anti-mouse or Alexa 594 goat anti-rabbit antibody) and DAPI for another hour. Coverslips were loaded on slides and cell fluorescence staining was viewed with confocal microscopy (Carl Zeiss Vision, LSM510).

### Co-immunoprecipitation

Co-immunoprecipitation was carried out as previously described^26^. Briefly, cells were lysed with IP buffer (50 mM Tris HCl, 150 mM NaCl, 1% NP40, 5 mM NaF, 1 mM Na_3_VO_3_, 5 μg/ml aprotinin, 1 mM PMSF) on ice for 1 h.. After centrifugation at 12,000×*g* for 5 min, supernatants were collected and precleared with either anti-mouse IgG or anti-rabbit IgG agarose beads. Precleared supernatants were then incubated with appropriate primary antibody for 2 h and further with TrueBlot^TM^ anti-mouse Ig IP beads or TrueBlot^TM^ anti-Rabbit Ig IP beads (Rockland, Limerick, PA). After several washes with IP buffer, beads were boiled and subjected to western blot analysis. Antibodies used were obtained from the following sources and used at the indicated titer: Jab1 (#MA1-23244, Thermo Scientific, 2 μg), MK2 (#3042, Cell signaling, 1:100), c-Jun (#610327, BD Transduction Laboratories, 2 μg), Jab1 (#6895, Cell signaling, 1:100), and HA-tag (#sc-805, Santa Cruz Biotechnology, 2 μg),

### Western blot analysis

Cells were harvested using radio-immunoprecipitation assay buffer supplemented with protease and phosphatase inhibitors (Bimake, Houston, TX). Equal amounts of protein were loaded per lane into an SDS-PAGE followed by transferring onto a nitrocellulose membrane (BioRad). The blots were blocked with 5% nonfat dried milk followed by incubation in the respective antibodies. After several washes, membranes were incubated with appropriate secondary antibodies and imaged using either chemiluminescence or the LICOR Odyssey Infrared Imaging System (Lincoln, NE). Antibodies for western blot analysis were used at 1:1000 dilution unless specified otherwise. HA-Tag #sc-7392, Fra-1 #sc-183, ATF-2 #sc-187, GAPDH #sc-137179 were from Santa Cruz Biotechnology; anti-phosphor-MK2 #07-155 from Upstate Biotechnology; Myc-tag #2866, ERK #9102, phosphor-ERK #4376, p38^MAPK^ #9212, phosphor-p38^MAPK^ #4511, JUN #9165, MK2 #3042, Jab1 #9444, Cyclin D1 #2922, βActin #4967 were from Cell Signaling; FLAG #F3165 was from MilliporeSigma; uPA #395 and uPAR #3937 were from American Diagnostica used at 1:500 dilution; Anti-phosphorylated JAB1 (Ser^177^) pAb was commercially prepared by EZ Biolab (Carmel, IN) using synthesized AVVIDPTRTI(pS)AGKVN peptide and used at 1:500 dilution. All blots derive from the same experiment and were processed in parallel.

### Quantitative RT-PCR

Total RNA was collected using Trizol, treated with DNaseI and used to generate cDNA. Generated cDNA was subjected to qRT-PCR to measure cyclin D1, uPA, uPAR, and βActin mRNA levels. The levels of cyclin D1, uPA, and uPAR were standardized by comparing its Ct values to that of βActin. RT-qPCR primer sequences are CAGGAGAGGAAAGCATGGAG and TCGGGTGAAATAATGGTGGT for cyclin D1; TGACCCACAGTGGAAAACAG and TTGTCCTTCAGGGCACATC for uPA; GGTGACGCCTTCAGCATGA and CCCACTGCGGTACTGGACAT for uPAR; CCAGCTCACCATGGATGATG, and ATGCCGGAGCCGTTGTC for βActin.

### In vitro kinase assay

In vitro kinase assay was carried out as previously described^26^. Briefly, 5 μg of recombinant JAB1 or JAB1-S177A was incubated with 1 μg active MK2 (Millipore, Billerica, MA) and 50 μCi [γ-32P]-ATP (Perkin Elmer, Boston, MA) in kinase assay buffer (50 mM Tris, pH 7.4, 1 mM DTT, 1 mM EGTA, 100 mM NaCl) at 30 °C for 1 h. Reactions were boiled with protein sample buffer and electrophoresed on SDS-polyacrylamide gels. Gels were dried and phosphorylation was analyzed by BIO-RAD Personal Molecular Imager System FX (BIO-RAD, Hercules, CA).

### Chromatin immunoprecipitation

ChIP was performed as previously described^57^. Briefly, 1 × 10^7^ cells per immunoprecipitate were cross-linked for 10 min with Formaldehyde to a final concentration of 1% at room temperature and quenched for 5 min with Glycine to a final concentration of 125 mM glycine. Cells were washed thrice with cold PBS and cell pellets were resuspended in lysis buffer (0.5% SDS, 25 mM EDTA, 100 mM NaCl, 10 mM Tris-HCl, pH 8.0) containing protease inhibitors. Cell suspensions were sonicated 15 cycles (30 s pulse, 30 s cooling) using a Bioruptor Pico (Diagenode, Denville, NJ). Cellular debris was eliminated by centrifugation at 10,000×*g* for 2 min. Supernatants were gently rotated overnight at 4 °C with 5 μg of rabbit IgG or RP-II or JUN pAb. After overnight incubation, immunocomplexes were transferred to tubes containing pre-washed Protein G Dynabeads (Life Technology) and incubated with gentle rotation at 4 °C for 3 h. Dynabeads-bound immunocomplexes were washed for 15 min each sequentially with low salt washing buffer (0.1% SDS, 1% TritonX-100, 2 mM EDTA, 150 mM NaCl, 20 mM Tris-HCl, pH 8.0), high salt washing buffer (0.1% SDS, 1% TritonX-100, 2 mM EDTA, 500 mM NaCl, 20 mM Tris-HCl, pH 8.0), and LiCl washing buffer (0.25 M LiCl, 1% NP-40, 1% sodium deoxycholate, 1 mM EDTA, 10 mM Tris-HCl, pH 8.0) at 4 °C. After washing, immunocomplexes were eluted with 500 μL elution buffer (1% SDS in 0.1 M NaHCO3) for 15 min at room temperature. Elutes were reverse cross-linked by incubation with 20 μL 5 M NaCl overnight at 65 °C. Next day, bound proteins in elutes were digested by incubation with digestion mix of 10 μL 0.5 M EDTA, 40 μL 1 M Tris-HCl pH 7.0, and 1 μL of 20 mg/ml Proteinase K at 45 °C for 2 h. DNA was extracted from digests with Phenol/Choloroform/Isoamyl alcohol method. Purified DNA were used to assess transcription (RP-II) and interaction of JUN-AP1 site. Promoter sequence enrichment was determined by qPCR using primers amplifying region either near the transcription start site or spanning AP1 site of the promoter. RP-II ChIP primer sequences are TCTGCCGGGCTTTGATCTTTG and TTGCAACTTCAACAAAACTCCCC for cyclin D1; CAAATCTTTGTGAGCGTTGCG and TCTCCGACTGTGCTGCGAC for uPA; AAGGGAAGTTTGTGGCGGAG and ACTCCTCCCAGACGTTTTGC for uPAR. JUN ChIP primer sequences are GGAACCTTCGGTGGTCTTGTC and GAATGGAAAGCTGAGAAACAGTGA for cyclin D1; TGTCACGCTTCATAACGGTCTC and CCCTAGCAGCTTTCATGACTC for uPA; and TCGAGGAATTCGAGAAGGAAC and TCCCGGAGCTGTTACTCATTC for uPAR.

### Orthotopic mouse xenograft model, imaging, and drug administration

Animal studies were performed as previously described^57^ and were approved by University of Florida Institutional Animal Care and Use Committee. Briefly, luciferase-expressing MDA-MB-231 cells were transduced with lentiviral vector containing either scramble (control) or JAB1 shRNA. Transduced cells were then injected into 4th left mammary fat pad of 5-week-old female nude mice (Jackson Lab, Bar Harbor, ME) at 3 × 10^6^ cells per mouse (5 mice each group) and tumor outgrowth was monitored weekly by measuring fluorescence in Xenogen IVIS-200 In Vivo imaging system (PerkinElmer Inc, Waltham, MA) for 4 weeks. In a parallel group, luciferase-expressing MDA-MB-231 cells were similarly injected. After 5 days of tumor cell injections, mice were randomized into two groups (5 per group) and treated daily with either vehicle or 1 µmol/kg of SB203580 i.p. for 4 weeks. Tumor growth were monitored weekly though Xenogen IVIS-200 in vivo imaging system.

### Bioinformatics analysis

JAB1 expression in different breast cancer subtypes were downloaded from https://tcga.xenahubs.net/download/TCGA.BRCA.sampleMap/HiSeqV2.gz, and https://tcga.xenahubs.net/download/TCGA.BRCA.sampleMap/BRCA_clinicalMatrix.gz. JAB1 mRNA levels were compared between TNBC and luminal subtypes or normal-like subtype. Statistical analysis was performed using GraphPad Prime 7.0 with unpaired *t* test (two-tailed). Kaplan–Meier curves were generated using http://kmplot.com/analysis/index.php?p=service&cancer=pancancer_rnaseq. Association between JAB1 and TNBC survival was analyzed by Cutoff^58^ and detailed codes are available at https://github.com/yueli8?COPS5_cutoff. For analysis of p38^MAPK^ activity in breast cancer, Reverse Phase Protein Array (RPPA) *z* score and corresponding clinical data from TCGA Breast Cancer Invasive Carcinoma, PanCancer Atlas were first downloaded through cBioportal (https://www.cbioportal.org/). Phospho-p38a level (MAPK14 pT180/Y182) levels were compared between basal and luminal subtypes. Statistical analysis was performed using GraphPad Prime 7.0 with unpaired *t* test (two-tailed).

### Statistical analysis

All experiments were performed in triplicate. The results of each experiment are reported as the mean of experimental replicates. Error bars represent the Standard Deviation (SD), unless otherwise stated. Statistical analyses of cell growth, in vitro invasion, mRNA levels and tumor development were performed by ANOVA and student *t* tests. Pairwise comparisons were analyzed using the unpaired *t* test with Welch’s correction (not assuming for equal SDs) to determine significance between control and knockdown. For all tests, *p* < 0.05 was considered significant.

### Reporting summary

Further information on research design is available in the Nature Research Reporting Summary linked to this article.

## Supplementary information

Supplementary Materials

Reporting Summary

## Data Availability

The data generated and analyzed during this study are described in the following data record: 10.6084/m9.figshare.14681250^59^. Files underlying the figures are openly available in Excel format with this data record. All other data supporting the study can be found in the supplementary information file, and the corresponding author can make any materials available upon request. Un-cropped gels and western blots for Fig. 1 to Fig. 5 were included in supplementary materials (Fig. S11). JAB1 expression in different breast cancer subtypes were downloaded from https://tcga.xenahubs.net/download/TCGA.BRCA.sampleMap/HiSeqV2.gz, and https://tcga.xenahubs.net/download/TCGA.BRCA.sampleMap/BRCA_clinicalMatrix.gz. For analysis of p38MAPK activity in breast cancer, Reverse Phase Protein Array (RPPA) *z* score and corresponding clinical data from TCGA Breast Cancer Invasive Carcinoma, PanCancer Atlas were first downloaded through cBioportal (https://www.cbioportal.org/).
