# Supplementary material for: Signaling of MK2 sustains robust AP1 activity for triple negative breast cancer tumorigenesis through direct phosphorylation of JAB1
Source: NPJ Breast Cancer. 2021 Jul 9;7:91. doi: 10.1038/s41523-021-00300-1 (PMC8270897; doi:10.1038/s41523-021-00300-1)
Supplement: Supplementary file 1 — Supplementary Materials [file 41523_2021_300_MOESM1_ESM.pdf]

SUPPLEMENTARY MATERIALS

Signaling of MK2 Sustains Robust AP1 Activity for Triple Negative Breast Cancer Tumorigenesis through Direct Phosphorylation of JAB1

Haoming Chen, Ravi Padia, Tao Li, Yue Li, Bin Li, Lingtao Jin, Shuang Huang

CORRESPONDENCE TO:
shuanghuang@ufl.edu

This PDF file includes:

FIGURES. S1 TO S11
TABLES S1

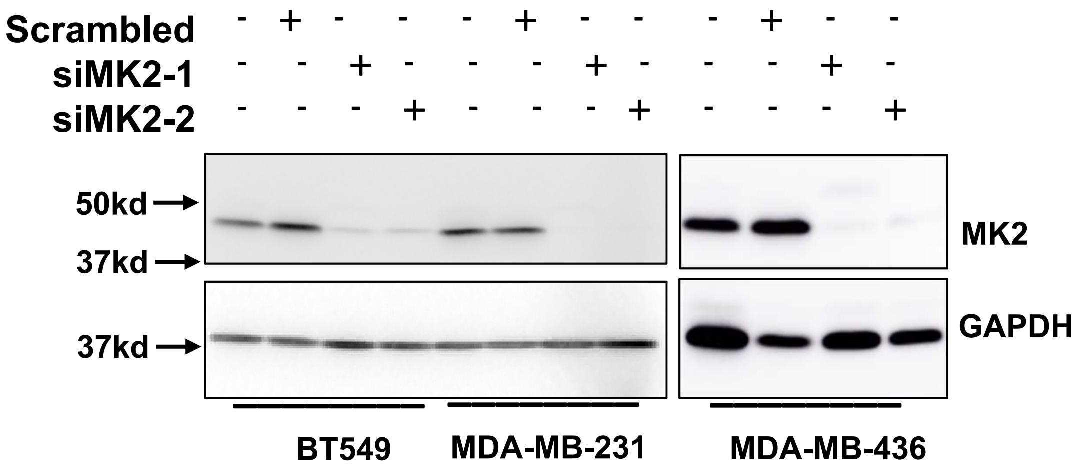

Supplementary Figure 1. Knockdown of MK2 by MK2 siRNAs in TNBC cell lines. Overnight-cultured cells were transfected with or without scrambled or MK2 siRNAs for 3 days followed by western blot analysis to detect MK2. Membranes were stripped and reprobed with anti-GAPDH polyclonal antibody for loading control.

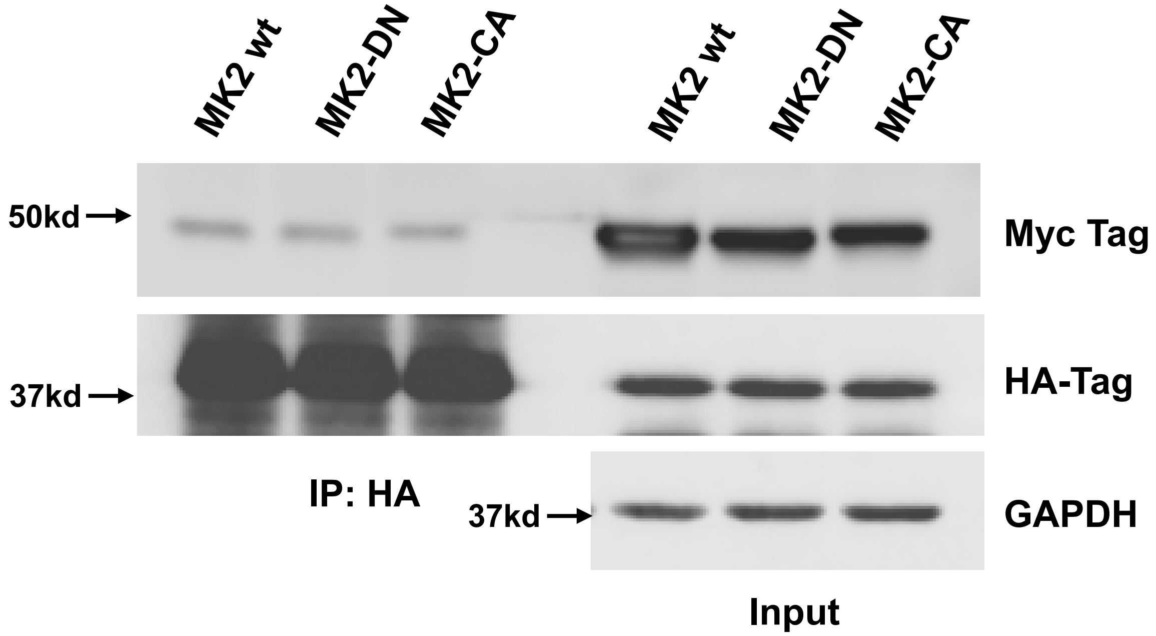

Supplementary Figure 2 JAB1-Mk2 interaction is not affected by MK2 activation status. HEK293 cells were co-transfected with Myc-tagged MK2 and HA-tagged JAB1 overnight. Cells were lysed and lysates were immunoprecipitated with HA pAb followed by western blotting to detect Myc-tagged MK2 using Myc Tag mAb. IP: immunoprecipitation. Input: cell lysate.

a

Sample 13916 (Jab1)

The target protein, CSN5_HUMAN, was identified with 82% sequence coverage:

sp|Q92905|CSN5_HUMAN (100%), 37,579.9 Da
COP9 signalosome complex subunit 5 OS=Homo sapiens GN=COPS5 PE=1 SV=4
42 unique peptides, 119 unique spectra, 267 total spectra, 275/334 amino acids (82% coverage)

```
MAASGSGMAQ KTWELANNMQ EAQSIDEIYK YDKKQQQEIL AAKPWTKDHH YFKYCKISAL
ALLKMVMHAR SGGNLEVMGL MLGKVDGETM IIMDSFALPV EGTETRVNAQ AAAYEYMAAY
IENAKQVGRL ENAIGWYHSH PGYGCWLSGI DVSTQMLNQQ FQEPFVAVVI DPTRTISAGK
VNLGAFRTYP KGYKPPDEGP SEYQTIPLNK IEDFGVHCKQ YYALEVSYFK SSLDRKLLEL
LWNKYWVNTL SSSSLLTNAD YTTGQVFDLS EKLEQSEAQL GRGSFMLGLE THDRKSEDKL
AKATRDSCKT TIEAIHGLMS QVIKDKLFNQ INIS
```

b

There were four putative phosphorylation sites detected, the combined coverage map is shown below, with modified residues indicated in green:

sp|Q92905|CSN5_HUMAN (100%), 37,579.9 Da
COP9 signalosome complex subunit 5 OS=Homo sapiens GN=COPS5 PE=1 SV=4
4 unique peptides, 6 unique spectra, 6 total spectra, 57/334 amino acids (17% coverage)

```
MAASGSGMAQ KTWELANNMQ EAQSIDEIYK YDKKQQQEIL AAKPWTKDHH YFKYCKISAL
ALLKMVMHAR SGGNLEVMGL MLGKVDGETM IIMDSFALPV EGTETRVNAQ AAAYEYMAAY
IENAKQVGRL ENAIGWYHSH PGYGCWLSGI DVSTQMLNQQ FQEPFVAVVI DPTRTISAGK
VNLGAFRTYP KGYKPPDEGP SEYQTIPLNK IEDFGVHCKQ YYALEVSYFK SSLDRKLLEL
LWNKYWVNTL SSSSLLTNAD YTTGQVFDLS EKLEQSEAQL GRGSFMLGLE THDRKSEDKL
AKATRDSCKT TIEAIHGLMS QVIKDKLFNQ INIS
```

c

The following table lists the phosphorylated spectra:

Site	Sequence	Mascot Ion score	Modifications	Observed	Actual Mass	Charge	Delta PPM	Start	Stop
S24	(K)TWELANNMQEAQsIDEIYKYDKK(Q)	30.4	Phospho (+80)	966.4382	2,896.29	3	-2.285	12	34
S24	(K)TWELANNmQEAQsIDEIYKYDKK(Q)	27.18	Oxidation (+16), Phospho (+80)	971.7712	2,912.29	3	-0.7979	12	34
S177	(R)TIsAGKVNLGAFR(T)	49.27	Phospho (+80)	707.3658	1,412.72	2	-0.4763	175	187
S177	(R)TIsAGKVNLGAFR(T)	42.64	Phospho (+80)	471.9131	1,412.72	3	-0.2542	175	187
Y189	(K)VNLGAFRTyPK(G)	18.35	Phospho (+80)	449.2267	1,344.66	3	-0.7353	181	191
S320	(K)TTIEAIHGLMsQVIKDK(L)	32.95	Phospho (+80)	655.3353	1,962.98	3	-0.5802	310	326

Supplementary Figure 3. Mass spectrometry analysis of the MK2-dependent phosphorylation of JAB1. ***a.*** Amino acid sequence of p68. ***b***. Mass spectrometry analysis of gel-recovered recombinant Jab1 protein included 42 unique peptides, 119 unique spectra, 267 total spectra, and 275 of 334 amino acid residues (82% coverage). There were four putative phosphorylation site: Ser^{24}, Ser^{177}, Y^{189} and S^{320} (marked in green). ***c***. The phosphorylated spectra.

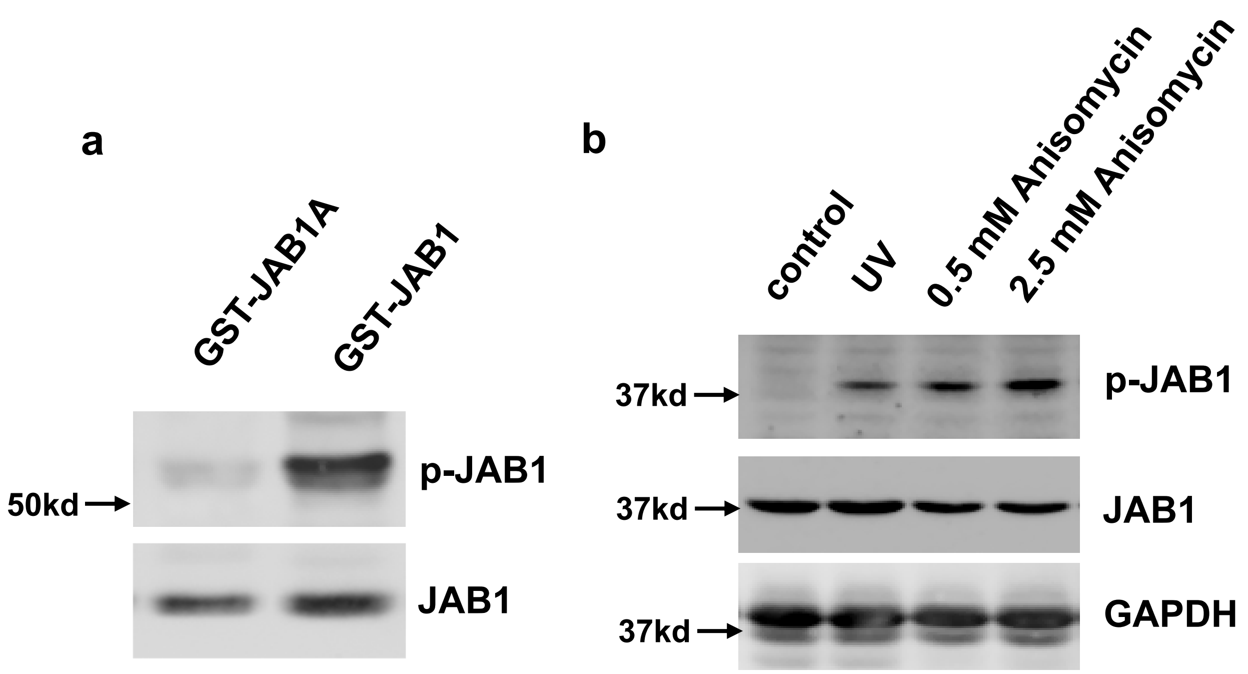

Supplementary Figure 4. Only wild-type recombinant JAB1 can be effectively phosphorylated by active MK2. ***a.*** Recombinant GST-JAB1 or JAB1A was incubated with active MK2 in presence of ATP for 30 min. Reaction mixtures were electrophoresed and transferred to nitrocellulose membrane. Membrane was blotted for anti-phosphor-JAB1 polyclonal antibody, Membrane was stripped and reprobed with JAB1 mAb. ***b***. MCF7 cells were either exposed to UV for 30 sec or treated with anisomycin or left untreated for 1 h followed by western blot to detect p-JAB1, JAB1 and GAPDH.

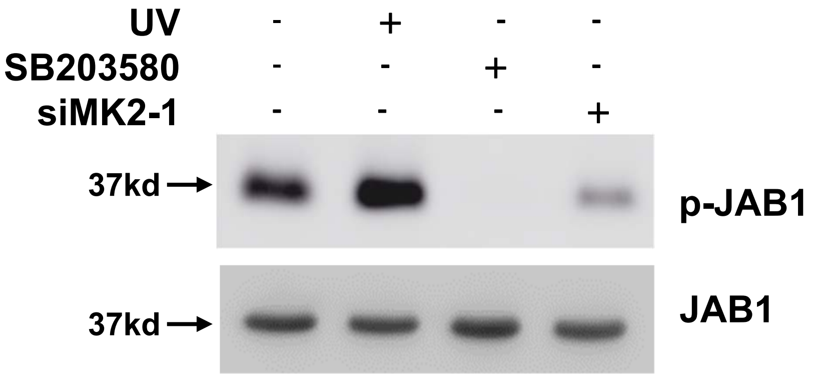

Supplementary Figure 5. Ser177 of JAB1 is associated with p38-MK2 signaling axis. MDA-MB-436 cells were under UV exposure for 1 min, treated with 5μM SB203580 for 1 days, 30nM MK2 siRNA (siMK2-1) for 3 days or left untreated followed by western blot analysis to detect Ser177-phosphorylated JAB1 and JAB1. Data are the representative of two independent experiments.

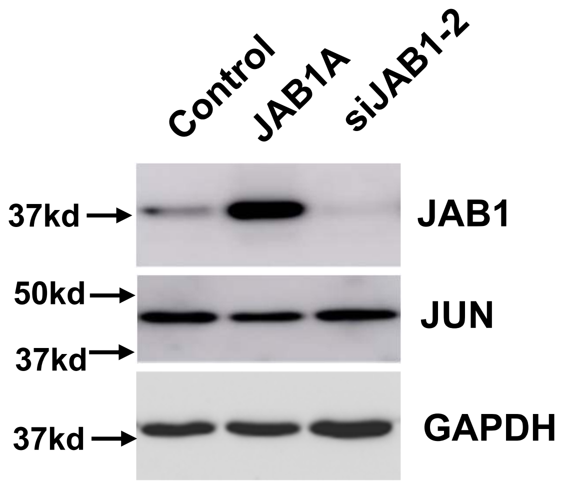

Supplementary Figure 6. JUN abundance is not affected by JAB1 presence or JAB1 phosphorylation status. MDA-MB-231 cells were lentivirally transduced with either JAB1 shRNA or JAB1A followed by western blotting to detect JUN with JUN mAb. Blot was stripped and reprobed with JAB1 and GAPDH antibodies.

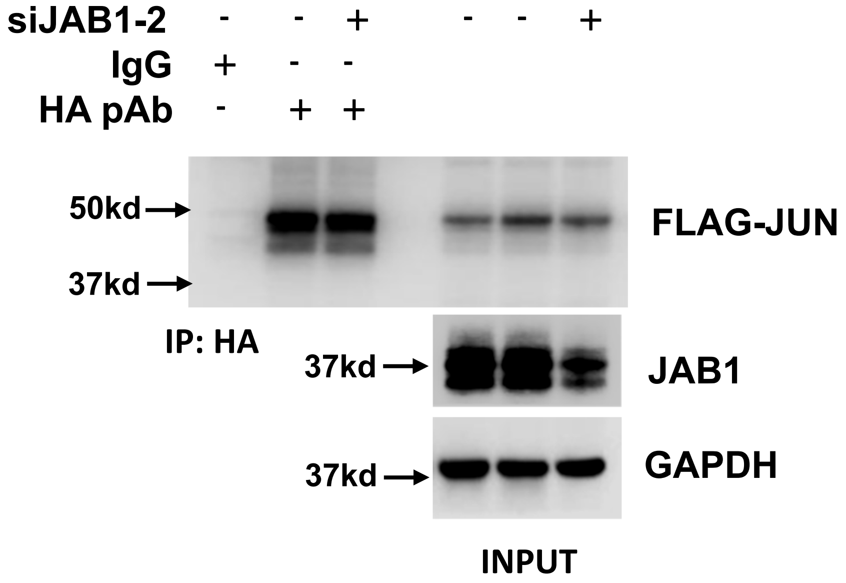

Supplementary Figure 7. Homo-dimerization of JUN is not affected by JAB1 depletion. Both HA-JUN and FLAG-JUN were lentivirally introduced into MDA-MB-231 cells and then either treated with scrambled siRNA control or JAB1 siRNA (siJAB1-2) for 3 days. Cells were lysed and cell lysates were immunoprecipitated with HA polyclonal antibody. Immuniprecipitates were subjected to western blot to detect FLAG-JUN with FLAG mAb (M2).

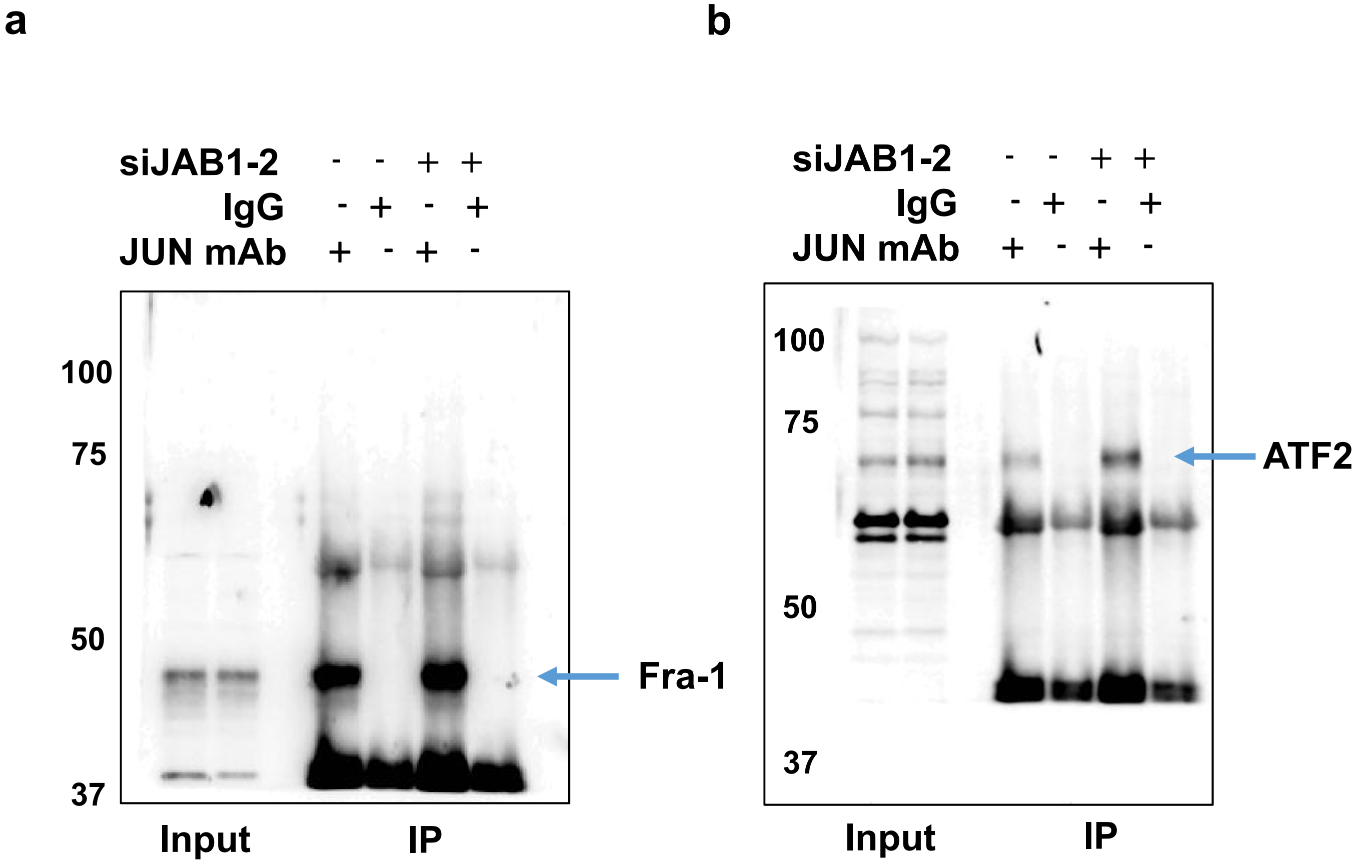

Supplementary Figure 8. Hetero-dimerization of JUN with Fra-1 or ATF2 is not affected by JAB1 depletion. MDA-MB-231 cells were treated with scrambled siRNA control or JAB1 siRNA (siJAB1-2) for 3 days. Cells were lysed and cell lysates were immunoprecipitated with JUN mAb or IgG. Immuniprecipitates were subjected to western blot to detect Fra-1 (***a***) or ATF2 (***b***).

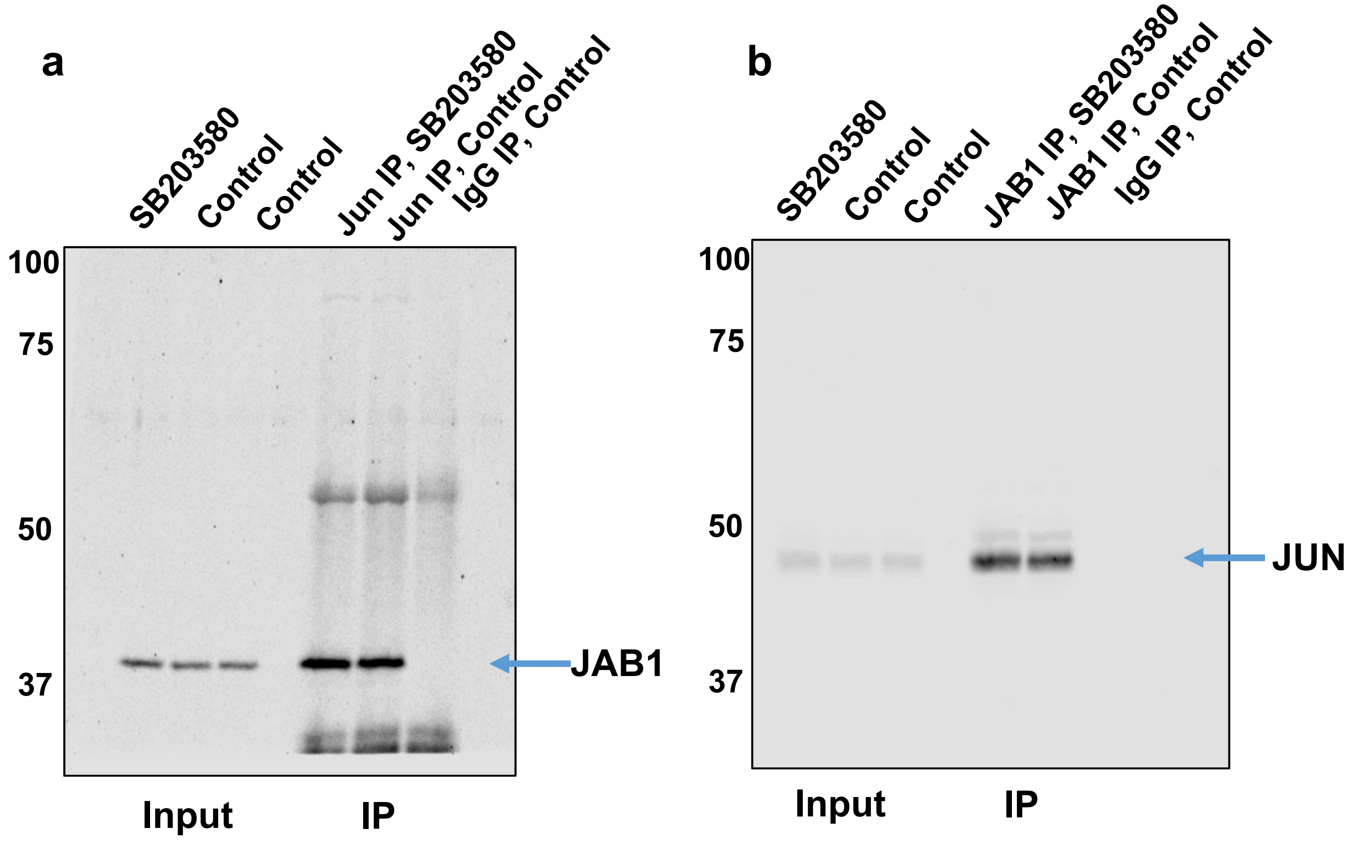

Supplementary Figure 9. Inhibiting p38 MAPK activity does not affect JUN-JAB1 interaction. MDA-MB-231 cells were treated with 10μM SB203580 for 6 h followed by immunoprecipitation with JUN, JAB1 mAb or IgG. Immunoprecipitates were subjected to western blot to detect JAB1 (***a***) or JUN (***b***).

Supplemental Figure S10

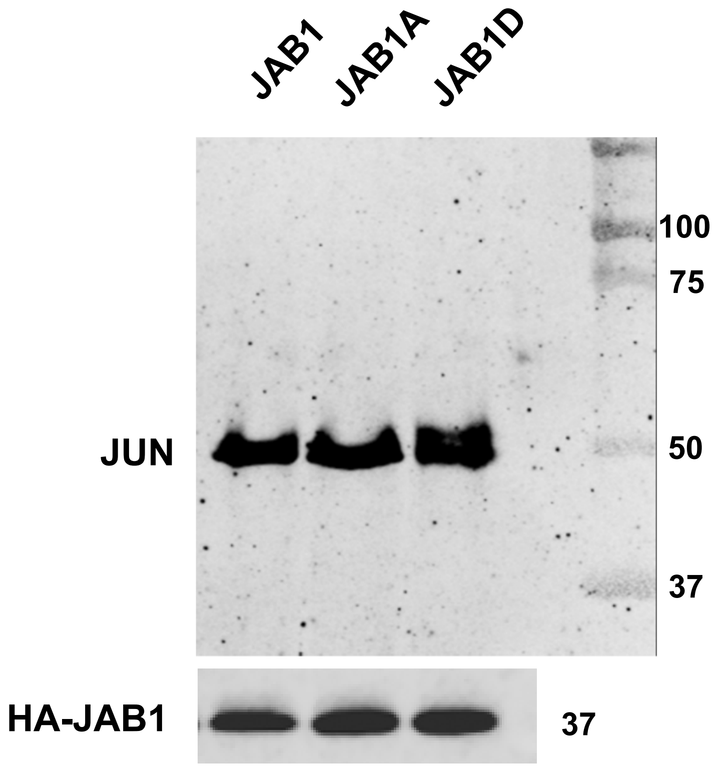

Supplementary Figure 10. Ser[177] phosphorylation status does not affect JAB1-JUN interaction. MDA-MB-231 cells were lentivirally transduced with HA-tagged JAB1, JAB1A or JAB1D, and then treated with 5μM SB203580 for 6 h followed by immunoprecipitation with HA pAb. Immunoprecipitates were subjected to western blot analysis to detect JUN with JUN mAb. Blot was stripped and reprobed with HA mAb.

Fig.1C

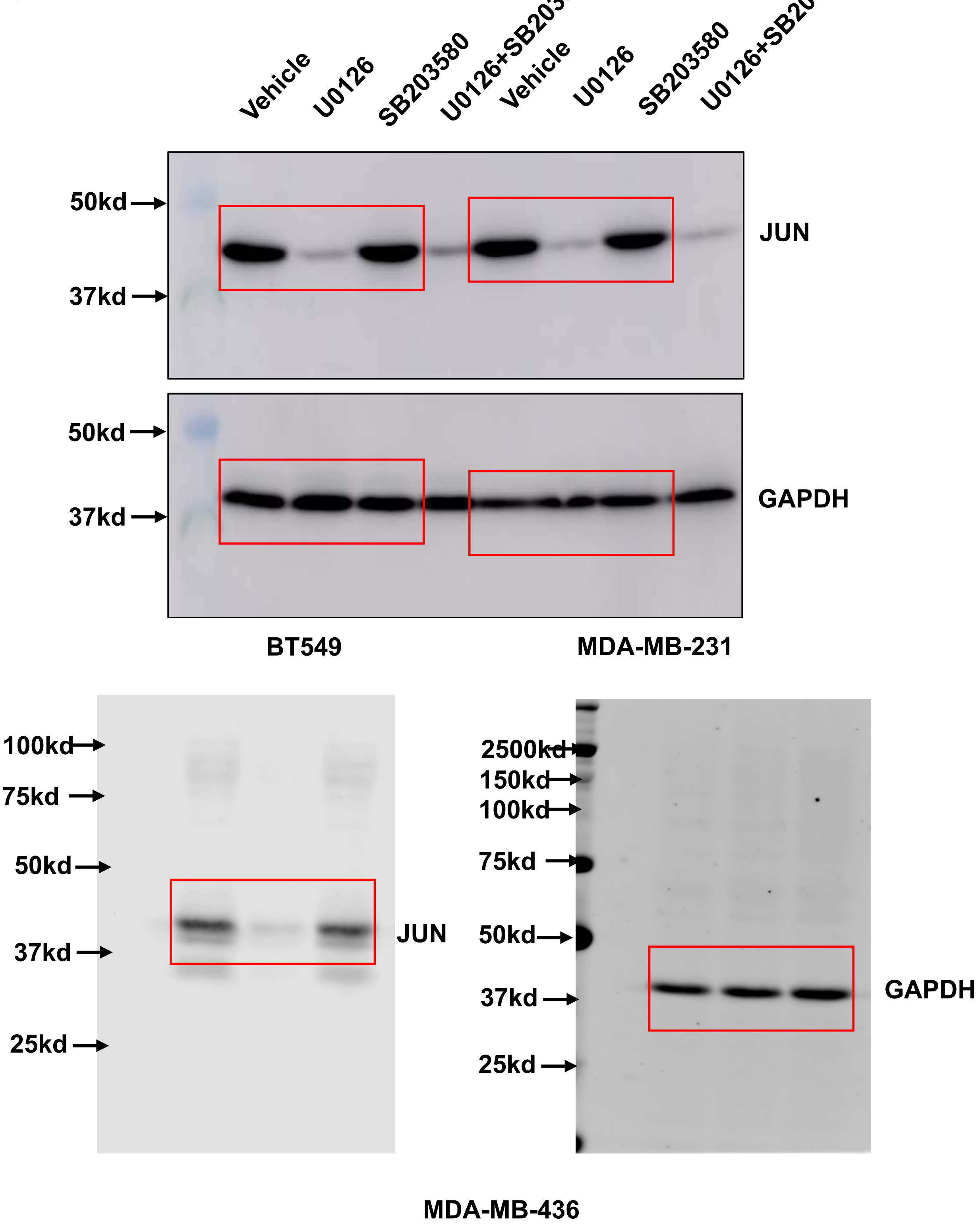

Vehicle
U0126
SB203580
U0126+SB203580
Vehicle
U0126
SB203580
U0126+SB203580
50kd
37kd
JUN
50kd
37kd
GAPDH
BT549
MDA-MB-231
100kd
75kd
50kd
37kd
25kd
JUN
2500kd
150kd
100kd
75kd
50kd
37kd
25kd
GAPDH
MDA-MB-436

Fig.2A

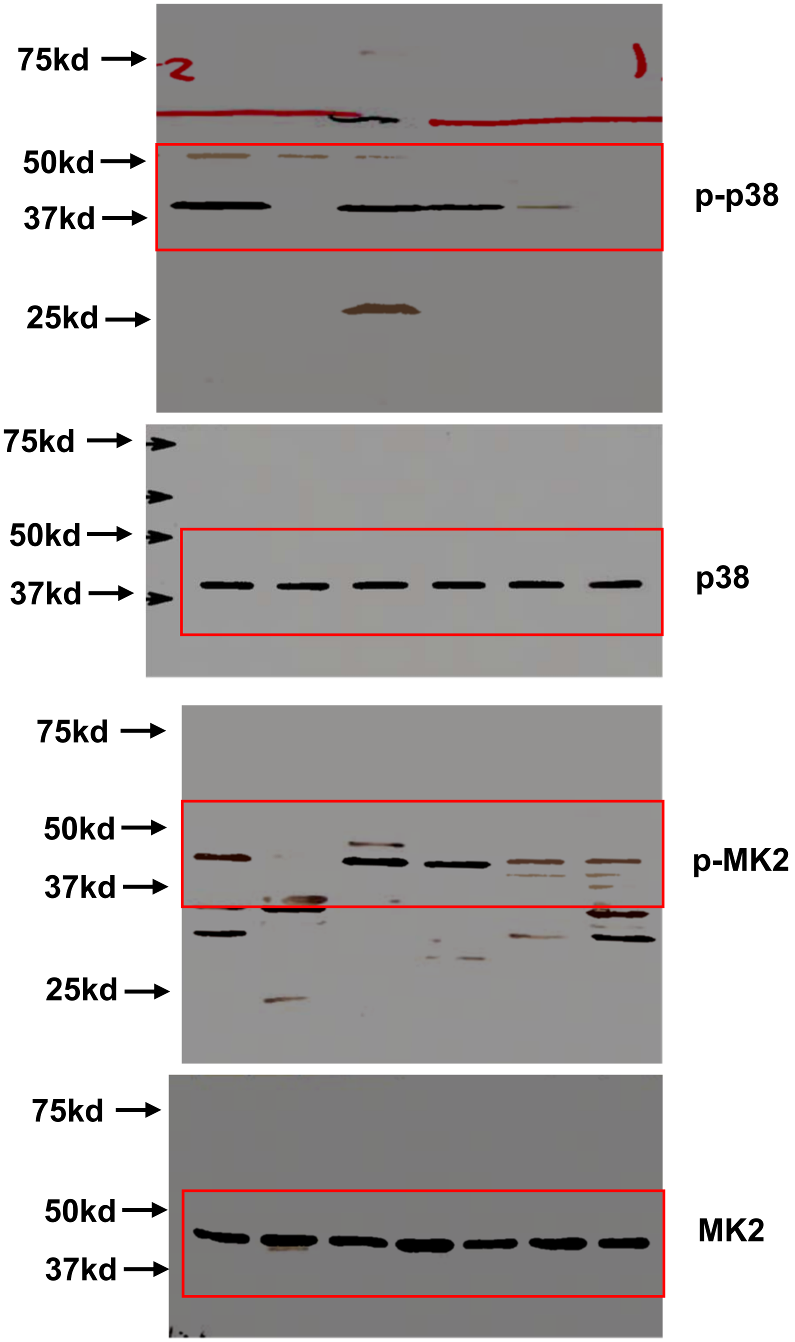

75kd
50kd
37kd
25kd
p-p38
75kd
50kd
37kd
p38
75kd
50kd
37kd
25kd
p-MK2
75kd
50kd
37kd
MK2

Fig.3A

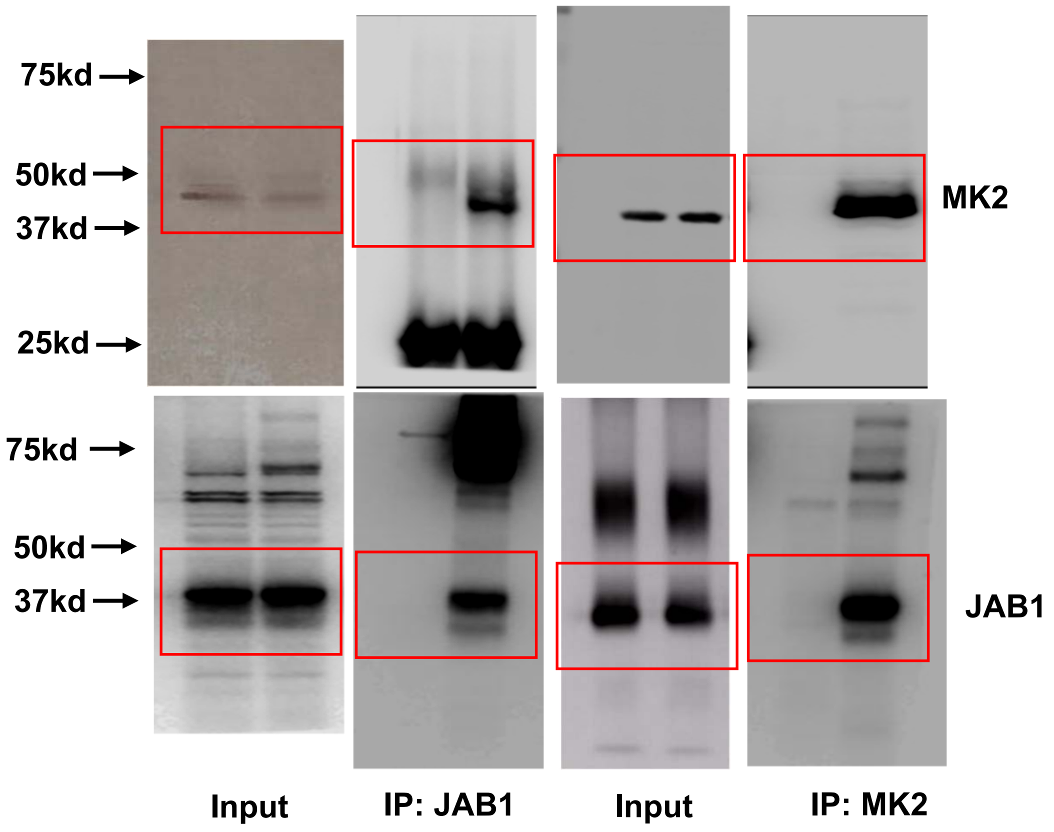
75kd
50kd
37kd
25kd
75kd
50kd
37kd
MK2
JAB1
Input
IP: JAB1
Input
IP: MK2

Fig.3D

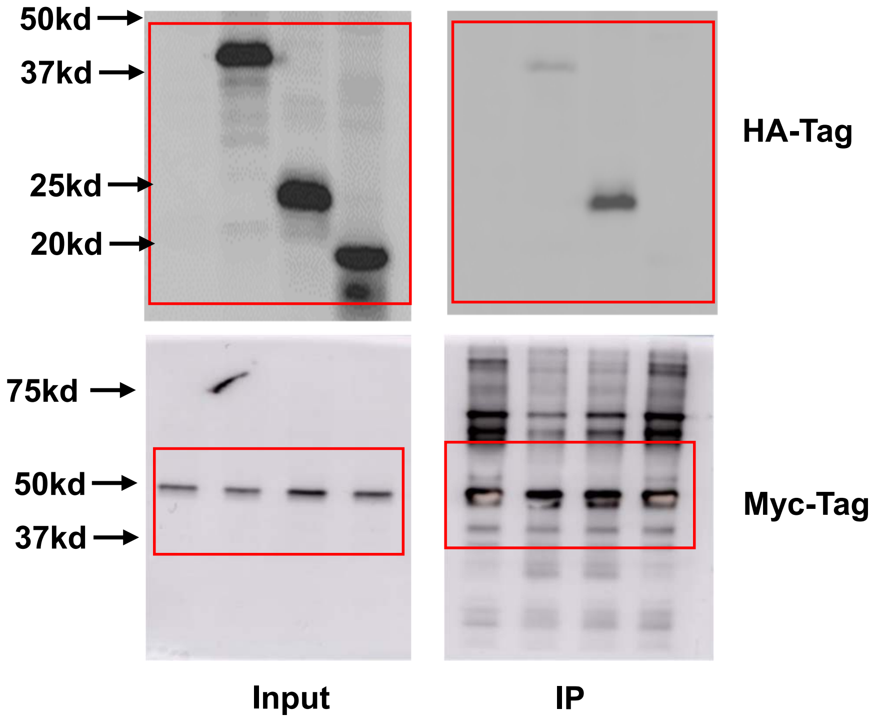
50kd
37kd
25kd
20kd
75kd
50kd
37kd
HA-Tag
Myc-Tag
Input
IP

Fig.4B

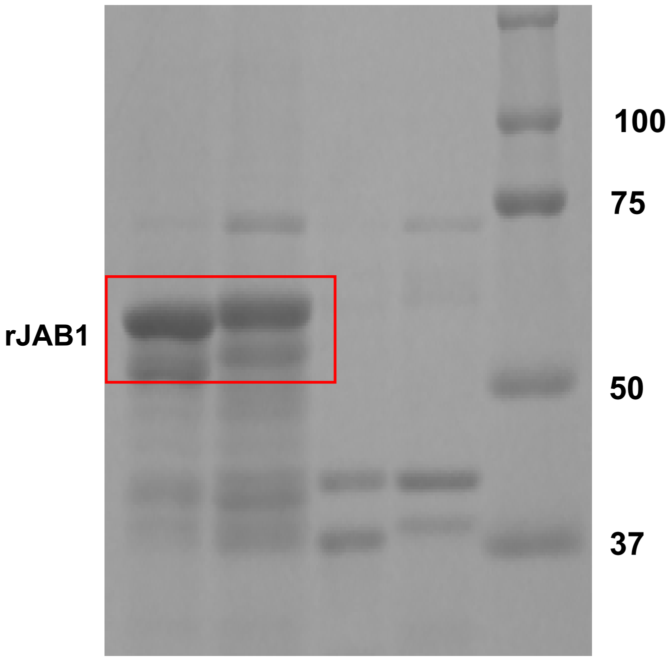

Fig.4C

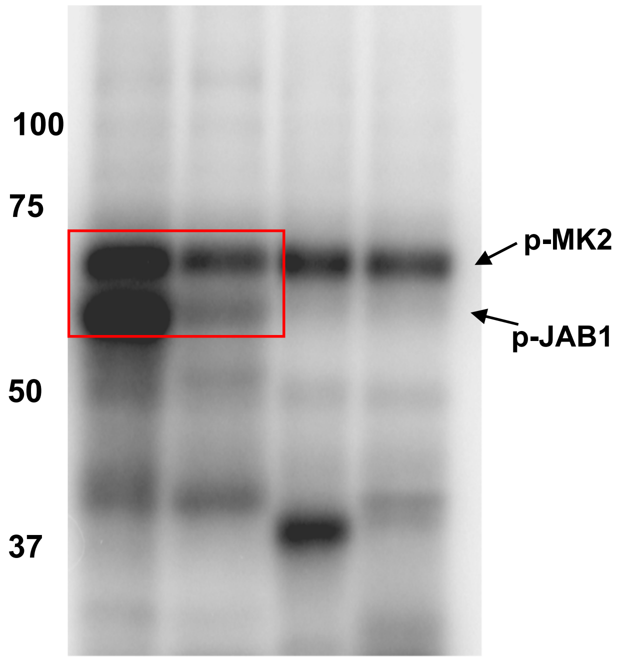

Fig.4D

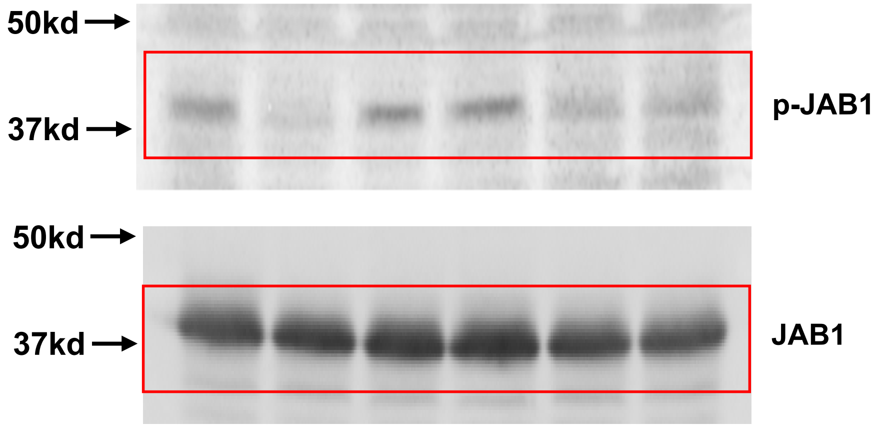

Fig.4E

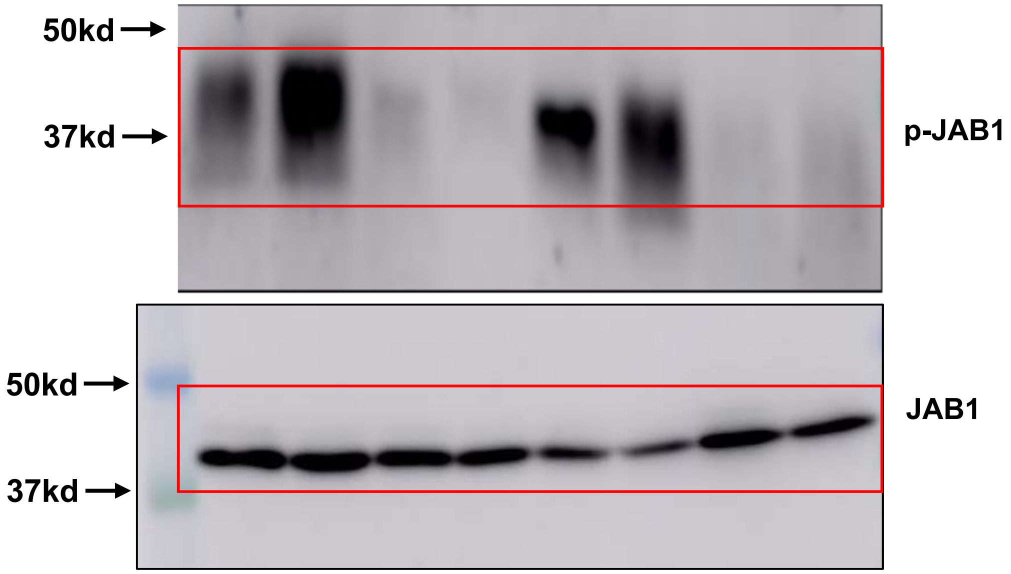

Fig.5A

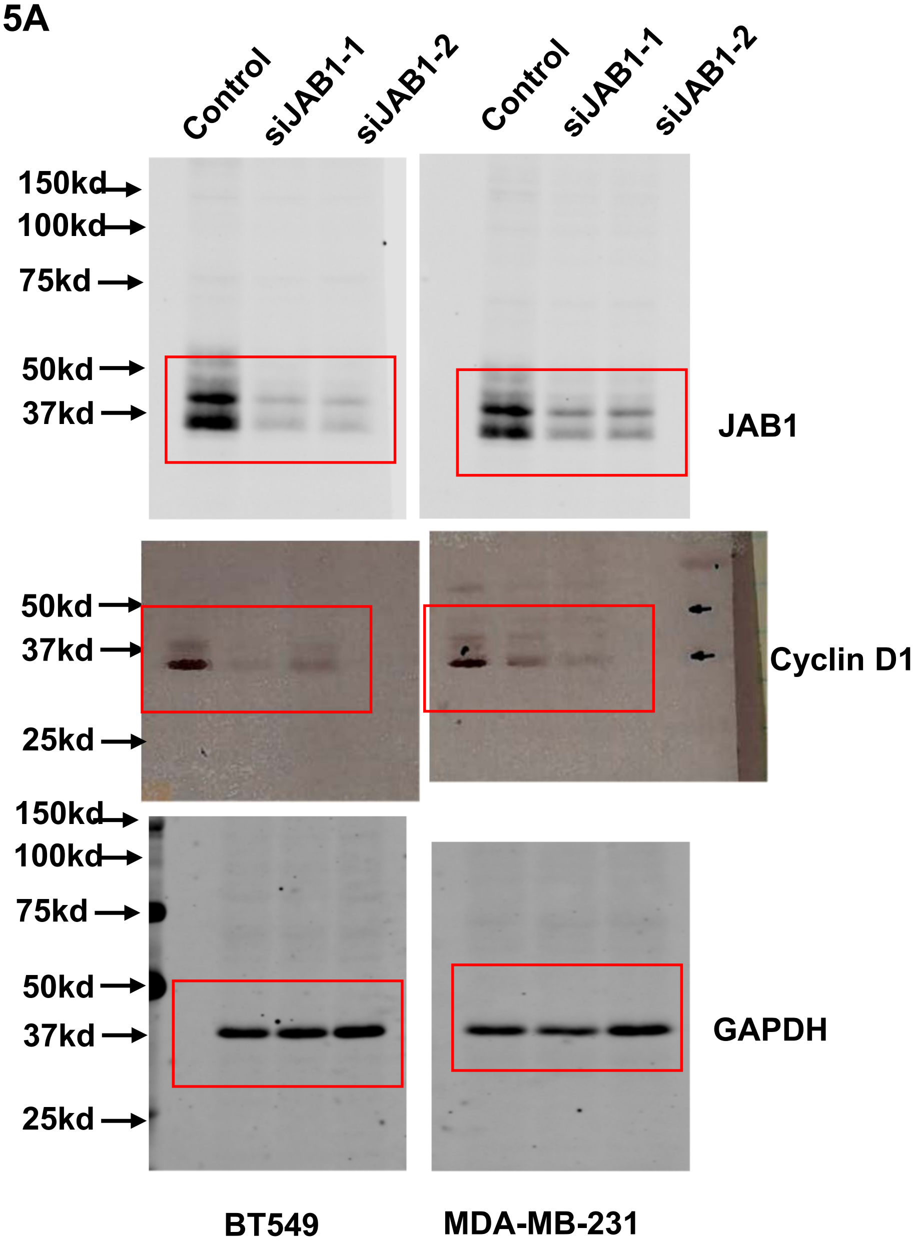
Control
siJAB1-1
siJAB1-2
Control
siJAB1-1
siJAB1-2
150kd
100kd
75kd
50kd
37kd
JAB1
50kd
37kd
25kd
Cyclin D1
150kd
100kd
75kd
50kd
37kd
25kd
GAPDH
BT549
MDA-MB-231

Fig.5A

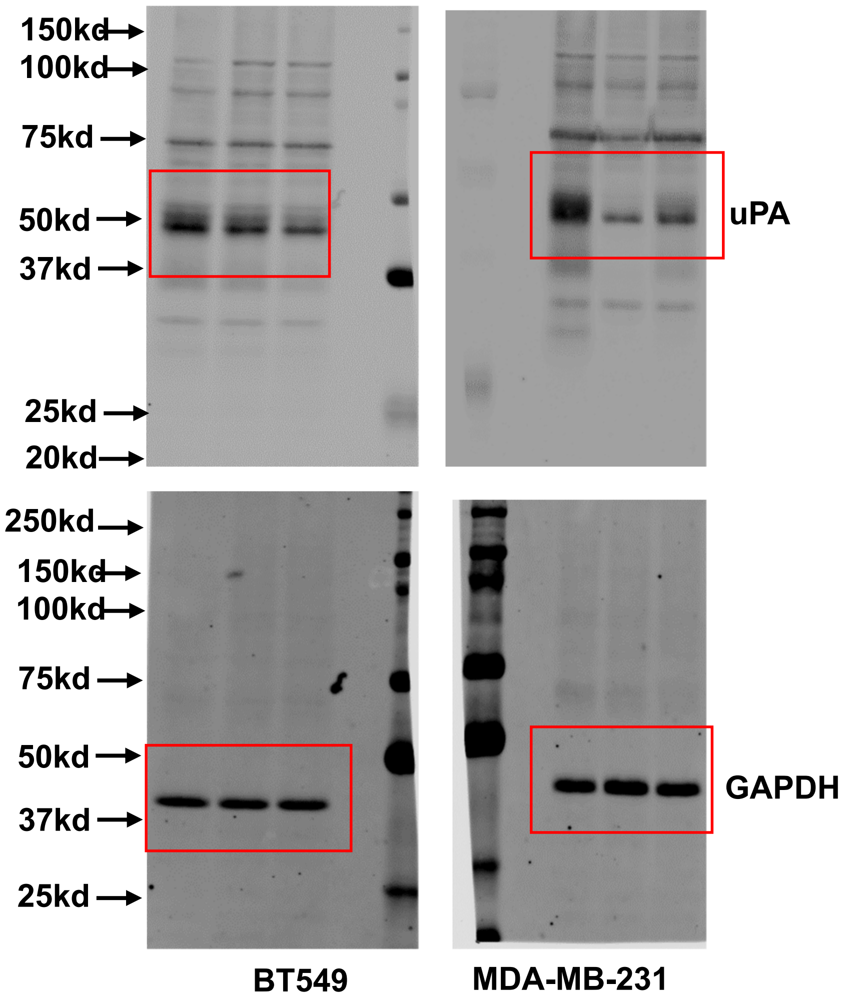

150kd
100kd
75kd
50kd
37kd
25kd
20kd
uPA
250kd
150kd
100kd
75kd
50kd
37kd
25kd
GAPDH
BT549
MDA-MB-231

Fig.5A

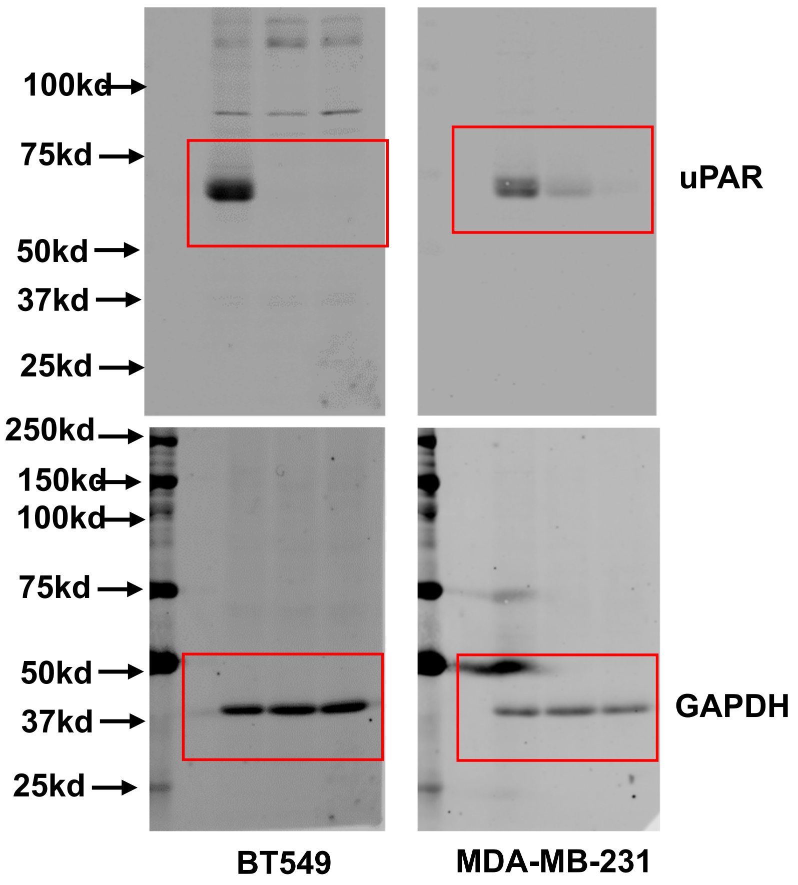

Supplementary Figure 11. Raw data for the uncropped blots included in Figure 1-5.

Supplementary Table 1: Correlation between JAB1 expression and clinical features (HiSeqV2)

**Clinical features**	COPS5 expression*		*p*-value**
	Low	High	
**Node**			
N0	330	55	0.004
N1, N2, N3	337	69	
**PANCAN_CNA_k8**			
High	254	67	0.000
COAD-READ, Squamous, BRCA_LUAD+, lq, kirc+, Quiet	486	65	
**Age_at_initial_Pathologic_Diagnosis**			
<=50	265	30	0.025
>50	538	100	
**Menopause_status**			
peri, pre	271	30	0.036
post	648	114	
**New_tumor_event_after_initial_treatment**			
NO	775	100	0.041
YES	87	20	
**Pathologic_Stage**			
I II	786	107	0.038
III IV	249	50	

*low/high are defined by cutoff finder. The expression level of COPS5 <=11.13 is low, while >11.13 is high. **Pearson's Chi-square test.
In https://tcga.xenahubs.net/download/TCGA.BRCA.sampleMap/HiSeqV2.gz, high COPS5 expression was correlated with node, PANCAN_CNA, age, menopause status, new tumor event after initial treatment and pathological stage (*P* = 0.004, 0.000, 0.025, 0.036, 0.041, 0.038).
