# Supplementary material for: Signaling of MK2 sustains robust AP1 activity for triple negative breast cancer tumorigenesis through direct phosphorylation of JAB1
Source: NPJ Breast Cancer. 2021 Jul 9;7:91. doi: 10.1038/s41523-021-00300-1 (PMC8270897; doi:10.1038/s41523-021-00300-1)
Supplement: Supplementary file 2 — Reporting Summary [file 41523_2021_300_MOESM2_ESM.pdf]

nature research

Corresponding author(s): Shuang Huang

Last updated by author(s): Jun 11, 2021

Reporting Summary

Nature Research wishes to improve the reproducibility of the work that we publish. This form provides structure for consistency and transparency in reporting. For further information on Nature Research policies, see our Editorial Policies and the Editorial Policy Checklist.

Statistics

For all statistical analyses, confirm that the following items are present in the figure legend, table legend, main text, or Methods section.

n/a	Confirmed	
☐	☒	The exact sample size (*n*) for each experimental group/condition, given as a discrete number and unit of measurement
☐	☒	A statement on whether measurements were taken from distinct samples or whether the same sample was measured repeatedly
☐	☒	The statistical test(s) used AND whether they are one- or two-sided<br>*Only common tests should be described solely by name; describe more complex techniques in the Methods section.*
☒	☐	A description of all covariates tested
☒	☐	A description of any assumptions or corrections, such as tests of normality and adjustment for multiple comparisons
☐	☒	A full description of the statistical parameters including central tendency (e.g. means) or other basic estimates (e.g. regression coefficient) AND variation (e.g. standard deviation) or associated estimates of uncertainty (e.g. confidence intervals)
☒	☐	For null hypothesis testing, the test statistic (e.g. *F*, *t*, *r*) with confidence intervals, effect sizes, degrees of freedom and *P* value noted<br>*Give P values as exact values whenever suitable.*
☒	☐	For Bayesian analysis, information on the choice of priors and Markov chain Monte Carlo settings
☒	☐	For hierarchical and complex designs, identification of the appropriate level for tests and full reporting of outcomes
☒	☐	Estimates of effect sizes (e.g. Cohen's *d*, Pearson's *r*), indicating how they were calculated

Our web collection on statistics for biologists contains articles on many of the points above.

Software and code

Policy information about availability of computer code

Data collection	TCGA data set, cbioportal
Data analysis	Microsoft Excel, Prism GraphPad, and Finding Cut-Point with R

For manuscripts utilizing custom algorithms or software that are central to the research but not yet described in published literature, software must be made available to editors and reviewers. We strongly encourage code deposition in a community repository (e.g. GitHub). See the Nature Research guidelines for submitting code & software for further information.

Data

Policy information about availability of data

All manuscripts must include a data availability statement. This statement should provide the following information, where applicable:

- Accession codes, unique identifiers, or web links for publicly available datasets
- A list of figures that have associated raw data
- A description of any restrictions on data availability

All the data support the findings in this study can be found within the article and its supplementary data. They also can be requested by contacting corresponding authors. Un-cropped gels and western blots for Fig.1 to Fig.5 were included in Supplementary Materials (Fig.S11).

Field-specific reporting

Please select the one below that is the best fit for your research. If you are not sure, read the appropriate sections before making your selection.

☒ Life sciences ☐ Behavioural & social sciences ☐ Ecological, evolutionary & environmental sciences

For a reference copy of the document with all sections, see nature.com/documents/nr-reporting-summary-flat.pdf

Life sciences study design

All studies must disclose on these points even when the disclosure is negative.

Sample size	No statistical method was used to predetermine sample size. The sample sizes were chosen based on our previous experience. All in vitro experiments were performed in triplicates and five animals were enrolled in each group for in vivo studies.
Data exclusions	No data exclusion
Replication	Most of in vitro experiments were repeated 3 times and a few done twice. All data presented in the manuscript were a representative and only reproducible results were included in the manuscript. All animal experiments were only done once and ICAUC from UF does not allow repeating the same experiment.
Randomization	Randomization is not applicable to all in vitro experiments. For all animal studies, animals were randomly chosen.
Blinding	Concealed allocation and blinding of outcome assessment were used.

Reporting for specific materials, systems and methods

We require information from authors about some types of materials, experimental systems and methods used in many studies. Here, indicate whether each material, system or method listed is relevant to your study. If you are not sure if a list item applies to your research, read the appropriate section before selecting a response.

Materials & experimental systems

n/a	Involved in the study	
☐	☒	Antibodies
☐	☒	Eukaryotic cell lines
☒	☐	Palaeontology and archaeology
☐	☒	Animals and other organisms
☒	☐	Human research participants
☒	☐	Clinical data
☒	☐	Dual use research of concern

Methods

n/a	Involved in the study	
☒	☐	ChIP-seq
☒	☐	Flow cytometry
☒	☐	MRI-based neuroimaging

Antibodies

Antibodies used	JAB1 monoclonal antibody (Catalog # MA1-23248, ThermoFisher)<br>MK2 polyclonal antobody (Catlog#3042, Cell Signaling)<br>HA-Tag antibody (Catalog# sc-7392, Santa Cruz Biotechnology)<br>uPA monoclonal antibody (Catalog# PIMA531100, ThermoFisher)<br>uPAR polyclonal antibody (Catalog# PIPA3001, ThermoFisher)<br>phosphor-MK2 polyclonal antibody (Catalog# SAB4503769, Millipore)<br>Myc-Tag monoclonal antibody (Catalog# 2276, Cell Signaling)<br>ERK mAb (Catalog# 4696, Cell Signaling)<br>phospho-ERK polyclonal antibody (Catalog# 9101, Cell Signaling)<br>p38 polyclonal antibody (Catalog# 9212, Cell Signaling)<br>phospho-p38 mAb (Catalog# 9216, Cell Signaling)<br>JUN Rabbit mAb (Catalog# 9165, Cell Signaling)<br>Cyclin D1 Rabbit mAb (Catalog# 2978, Cell Signaling)
Validation	All antibodies used were confirmed by the manufacturers for the experiments performed in this manuscripts. We also confirmed most of these antibodies by either overexpression or siRNA-mediated knockdown.

April 2020

Eukaryotic cell lines

Policy information about cell lines

Cell line source(s)	ATCC
Authentication	All lines are authenticated by institute's core facility for authenticity in yearly base
Mycoplasma contamination	Mycoplama is routinely checked using PCR-based method in the lab and only mycoplama-negative cells were used for the experiments performed in this manuscript.
Commonly misidentified lines (See ICLAC register)	N/A

Animals and other organisms

Policy information about studies involving animals; ARRIVE guidelines recommended for reporting animal research

Laboratory animals	Foxn1nu, female (Catalog# 007850, The Jackson laboratory)
Wild animals	N/A
Field-collected samples	N/A
Ethics oversight	University of Florida Institutional Animal Care and Use Committee

Note that full information on the approval of the study protocol must also be provided in the manuscript.
